# The Emergence and Representation of Knowledge about Social and Nonsocial Hierarchies

**DOI:** 10.1016/j.neuron.2012.09.035

**Published:** 2012-11-08

**Authors:** Dharshan Kumaran, Hans Ludwig Melo, Emrah Duzel

**Affiliations:** 1 Institute of Cognitive Neuroscience, University College London, 17 Queen Square, WC1N 3AR, UK; 2 Institute of Cognitive Neurology and Dementia Research, Otto-von-Guericke University, Magdeburg, Leipziger Strasse 44, 39120 Magdeburg, Germany; 3 German Center for Neurodegenerative Diseases, 39120 Magdeburg, Germany

## Abstract

Primates are remarkably adept at ranking each other within social hierarchies, a capacity that is critical to successful group living. Surprisingly little, however, is understood about the neurobiology underlying this quintessential aspect of primate cognition. In our experiment, participants first acquired knowledge about a social and a nonsocial hierarchy and then used this information to guide investment decisions. We found that neural activity in the amygdala tracked the development of knowledge about a social, but not a nonsocial, hierarchy. Further, structural variations in amygdala gray matter volume accounted for interindividual differences in social transitivity performance. Finally, the amygdala expressed a neural signal selectively coding for social rank, whose robustness predicted the influence of rank on participants’ investment decisions. In contrast, we observed that the linear structure of both social and nonsocial hierarchies was represented at a neural level in the hippocampus. Our study implicates the amygdala in the emergence and representation of knowledge about social hierarchies and distinguishes the domain-general contribution of the hippocampus.

## Highlights

► Amygdala activity tracks emergence of knowledge about a social hierarchy ► Amygdala gray matter volume predicts social transitivity performance ► Amygdala selectively codes the rank of individual people in the hierarchy ► Hippocampus supports domain-general representations of linear hierarchies

## Teaser

The study by Kumaran et al. provides insights how the amygdala supports the acquisition and use of knowledge about social hierarchies.

## Introduction

Primates have sophisticated cognitive abilities that enable individuals to meet the challenging pressures of living in large social groups (Byrne and Bates, 2010; Cheney and Seyfarth, 1990; Tomasello and Call, 1997). Foremost among these is the capacity to judge the relative rank of others, which enables individuals to select advantageous coalition partners, and avoid potentially injurious conflicts (Cheney and Seyfarth, 1990; Tomasello and Call, 1997). Two different sources of information may be used to guide judgments of social rank: first, the physical appearance of an individual (e.g., facial features and body posture: Karafin et al., 2004; Marsh et al., 2009; Todorov et al., 2008; Zink et al., 2008)—and second, specific knowledge of the hierarchical structure of the social group, accrued through experience of previous encounters and interactions (Byrne and Bates, 2010; Cheney and Seyfarth, 1990; Grosenick et al., 2007; Paz-Y-Miño et al., 2004). While perceptual cues may provide a useful, but relatively imprecise, heuristic with which to rapidly evaluate an unfamiliar individual (e.g., an intruder: Marsh et al., 2009; Todorov et al., 2008; Whalen, 1998), specific knowledge of the rank position of a fellow group member is needed to support more accurate judgments of rank (Cheney and Seyfarth, 1990; Tomasello and Call, 1997). Indeed, considerable evidence indicates that humans and nonhuman primates possess such knowledge, and are able to rank each other within linear hierarchies that are stable over long periods of time (Byrne and Bates, 2010; Cheney and Seyfarth, 1990; Savin-Williams, 1990). For instance, primates spontaneously discriminate images of individuals based on their rank status (Deaner et al., 2005) and are able to identify third-party relations that exist between their companions—when engaged in a competitive interaction (e.g., a duel) individuals will typically recruit allies that outrank both themselves and their opponents (e.g., favoring the 3rd ranked individual over the fifth ranked) (Cheney and Seyfarth, 1990; Tomasello and Call, 1997).

According to psychological theories grounded in research in animals, individuals acquire knowledge about social hierarchies by experiencing encounters between pairs of conspecifics, with such dyadic interactions either being experimentally enforced (Grosenick et al., 2007; Paz-Y-Miño et al., 2004) or occurring through the course of natural behavior (Cheney and Seyfarth, 1990; Tomasello and Call, 1997). Notably, however, individuals must confront a thorny obstacle during learning: the number of possible dyadic interactions scales exponentially with group size, thereby placing prohibitive demands on memory capacity (Byrne and Bates, 2010; Cheney and Seyfarth, 1990). Evidence suggests that individuals solve this problem in an elegant fashion—by restricting their observations to a small subset of all possible dyadic interactions, and then using a highly developed capacity for transitive inference to deduce the remaining rank relations between group members (i.e., if P1 > P2 & P2 > P3, then P1 > 3, where P1 denotes the highest ranking individual) (Byrne and Bates, 2010; Cheney and Seyfarth, 1990; Grosenick et al., 2007; Paz-Y-Miño et al., 2004). Indeed, it has been argued that the pressures of living in large social groups may have driven the evolution of sophisticated abilities for transitive inference, based on the finding that the more highly social of two closely related primate species exhibit superior capacities in this regard (e.g., Maclean et al., 2008).

Existing research in animals, therefore, has emphasized that rank judgments depend critically on knowledge about linear social hierarchies that is acquired through learning, and a highly developed capacity for transitive inference (Byrne and Bates, 2010; Cheney and Seyfarth, 1990; Grosenick et al., 2007; Paz-Y-Miño et al., 2004). Surprisingly little, however, is understood about the neurobiology underlying these core aspects of primate cognition. While previous work suggests that lesions to the amygdala in nonhuman primates (Kling, 1992; Machado and Bachevalier, 2006; Rosvold et al., 1954; although see Bauman et al., 2006) and the medial prefrontal cortex in mice (Wang et al., 2011) may cause affected individuals to fall in rank within the group, the role of these brain regions in representing knowledge about social hierarchies has not been investigated. In humans, previous fMRI studies have tended to investigate how status, a construct which relates broadly to rank, influences neural processing—where the status of an individual was well known to participants prior to the experiment (e.g., the Queen of England: Chiao et al., 2009; Farrow et al., 2011) or conveyed by perceptual cues (e.g., body posture, attire: Marsh et al., 2009; Zink et al., 2008). For instance, Zink et al. (2008) compared neural activity while participants viewed the face of a superior player, whose status was declared by the number of stars presented on the screen (e.g., three-star rating)—rather than learned through experience—with that of an inferior player (e.g., two-star rating). Based on existing evidence, therefore, the neural mechanisms by which knowledge about social hierarchies emerges through experience and is represented in the human brain remains a fundamental but open question in neuroscience.

**Figure 1:**
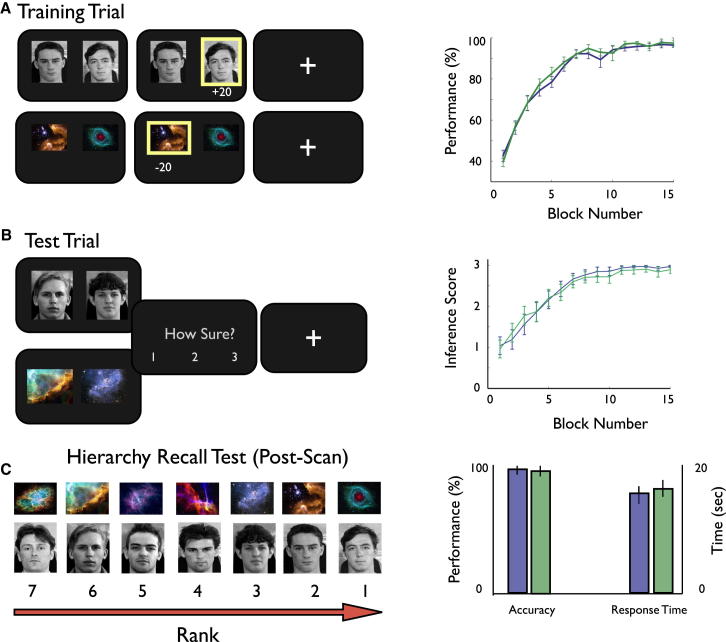
Learn Phase: Experimental Task and Behavioral Data (A) Training trials: timeline (left panel), behavioral data (right panel). Participants viewed adjacent items in the hierarchy (P1 P2, G1, G2 illustrated, where P1 = person of rank equal to 1, and G1 = galaxy of rank equal to 1) and selected the item which they thought had more power (social) or more mineral (nonsocial). Right panel shows training trial performance across all 15 experimental blocks, averaged across all 6 training trial types (e.g., P1 versus P2, P2 versus P3, etc.) and participants (person condition: blue, galaxy condition: green, error bars reflect SEM). (B) Test trials: timeline (left panel), behavioral data (right panel). Participants viewed nonadjacent items in the hierarchy (P3 P6, G3 G6 illustrated), inferred the higher ranking item, and rated their confidence in their choice—no feedback was provided. Right panel shows inference score index over all 15 experimental blocks, averaged across all 6 test trial types (e.g., P2 versus P4, P2 versus P5, etc.), and participants (person condition, blue; galaxy condition, green; error bars reflect SEM). The inference score index (range 0–3) was derived by combining (i.e., multiplying) the correctness of participants’ choices during test trials with their confidence rating, and indexed the level of hierarchical knowledge attained at a given time point during the Learn phase (see Supplemental Results). (C) Hierarchy recall test (debriefing session): pictures of the set of people and galaxies were presented to participants, and they were asked to rank them in terms of their order in the hierarchy, with their performance timed. Example hierarchies are illustrated—note the allocation of person and galaxy to rank position (1 = high rank, 7 = low rank) was randomized across participants. Right panel (person condition, blue; galaxy condition, green) shows performance (%) on hierarchy recall test, and time taken (seconds). Error bars reflect SEM.

To address these issues, we employed a two-phase experimental scenario, in combination with both functional (fMRI) and structural (voxel-based morphometry—VBM) neuroimaging techniques. In the first (“Learn”) phase, we used an experimental paradigm whose design was motivated by the acknowledged importance of learning and transitive behavior to social rank judgments (Cheney and Seyfarth, 1990; Grosenick et al., 2007; Paz-Y-Miño et al., 2004). Participants acquired knowledge about two seven-item hierarchies in parallel, whose emergence we could track at both behavioral and neural levels through online assessments of transitivity performance conducted across this experimental phase (see Supplemental Experimental Procedures available online). One hierarchy, herein termed social (c.f. Magee and Galinsky, 2008), comprised individual people in a fictitious company with different levels of power—the other, herein termed nonsocial, comprised galaxies with different levels of a precious mineral (Figure 1). In the second (“Invest”) phase, participants were required to use the knowledge about hierarchies that they had acquired during phase 1, and evaluate the potential worth of individual people and galaxies to guide economic pricing decisions. Importantly, person and galaxy rank were orthogonalized by experimental design in this phase, enabling us to define how rank information is coded at a neural level, separately for each stimulus type. Our experiment, therefore, was specifically set up to define the neural mechanisms operating during the emergence of knowledge about social hierarchies, examine how rank information is coded in the brain, and dissociate the operation of social-specific from domain-general processes.

## Results and Discussion

### Phase 1: Learn

Participants completed training trials, where a pair of adjacent items in the hierarchy was presented (e.g., P1 versus P2, G1 versus G2, where P = person and G = galaxy; Figure 1A): they were required to learn through trial and error, which person had more power (social condition) or which galaxy had more mineral (nonsocial condition). Following each block of training trials, participants completed test trials where they were required to select the higher ranking of the two items presented (e.g., P3 versus P6, G3 versus G6; Figure 1B) and rate their confidence in their decision on a scale of 1 (guess) to 3 (very sure). Test trials differed from training trials in two critical ways: nonadjacent items in the hierarchy were presented during test trials (e.g., P3 versus P6), and no corrective feedback was issued. As such, participants were required to use transitive inference to deduce the correct item during test trials (e.g., P3, in a P3 versus P6 trial), by using knowledge of the underlying hierarchy (e.g., P1 > P2 > P3 > P4 > P5 > P6 > P7: see below). In contrast, participants could achieve proficient performance on training trials by simply memorizing the correct item in each pair (e.g., P1, in a P1 versus P2 trial).

While the Learn phase paradigm builds on a rich vein of research that has used the transitive inference task across species (Bryant and Trabasso, 1971; Dusek and Eichenbaum, 1997; Greene et al., 2006; Grosenick et al., 2007; Heckers et al., 2004; Hurliman et al., 2005; Moses et al., 2010; Paz-Y-Miño et al., 2004; Zeithamova et al., 2012), we incorporated several features designed to achieve the specific goals of our experiment: first, we interleaved blocks of training and test trials throughout the time course of the Learn phase in order to chart the development of successful transitive behavior. In contrast, previous fMRI studies have typically included test trials only at the very end of training (Greene et al., 2006; Heckers et al., 2004; Moses et al., 2010). Second, we incorporated a novel measure of test trial performance (i.e., “inference score”), which was validated in a separate behavioral experiment (see below and Supplemental Results). The inference score index - which incorporated participants’ assessment of their confidence in their choices, a metacognitive measure typically used to characterize medial temporal lobe dependent memory processes (e.g., (Eichenbaum et al., 2007)—allowed us to track the emergence of knowledge of the linear structure of the hierarchy, and thereby reveal the underlying neural mechanisms. Lastly, our paradigm was unique in affording a direct comparison of social (i.e., person) and nonsocial (i.e., galaxy) hierarchy learning under conditions where behavioral performance was well matched (Figure 1).

### Behavioral Data

Participants improved their performance on training trials and test trials over the course of the Learn phase: no significant differences were found between social and nonsocial conditions, either in terms of the correctness of choices or the distribution of confidence ratings during test trials (ps > 0.1; Figures 1A and 1B). By the end of this experimental phase, almost all (i.e., 25 out of 26) participants exhibited proficient transitive behavior, reflected by the inference score index—the one participant that performed poorly in both social and nonsocial domains was excluded from the fMRI analysis.

Several considerations indicate that successful transitive behavior in our experiment was driven primarily by relational (or declarative) knowledge of the hierarchy (i.e., P1 > P2 > P3… > P7) (Cohen and Eichenbaum, 1993; Smith and Squire, 2005), whose evolution we were able to track through the use of the inference score index. First, in our experiment participants developed near-ceiling levels of transitive performance in the context of relatively long (i.e., seven-item) hierarchies—while alternative (e.g., reinforcement-based procedural; Frank et al., 2003) mechanisms may underlie modest (e.g., 60% correct) performance in settings where shorter (i.e., five-item) hierarchies are involved (e.g., Greene et al., 2006), hierarchy knowledge is required to mediate the highly proficient transitive behavior we observed (e.g., Frank et al., 2003). Second, participants expressed robust knowledge of the two seven-item hierarchies in the postexperimental debriefing session that followed the end of phase 2. As such, participants performed near perfectly when asked to recall the order of items in both hierarchies, with no significant difference observed between social and nonsocial hierarchies, in terms of accuracy, or response time: both ps > 0.1 (Figure 1C). Third, in a separate behavioral study we found that the inference score index showed a robust correlation with participants’ knowledge of the hierarchy—as measured by a direct test (e.g., Smith and Squire, 2005)—even once the correctness of participants’ test trial (and training trial) responses had been partialled out (see Supplemental Results). These data, therefore, in demonstrating that the inference score index has objective explanatory value (c.f. the binary choice data alone), provide support for its use as a proxy for the level of hierarchical knowledge attained by a given participant over the time course of the Learn phase.

### Functional Neuroimaging (fMRI) Data

#### Neural Activity in the Amygdala/Anterior Hippocampus Selectively Tracks the Emergence of Knowledge about a Social Hierarchy

Given behavioral evidence that participants acquired knowledge about both social and nonsocial hierarchies over the course of the Learn phase, and furnished with an online index tracking its emergence, we next turned to fMRI data. We created participant-specific trial-by-trial parametric regressors that we used to regress against the test trial fMRI data, with separate regressors included for social (i.e., person) and nonsocial (i.e., galaxy) conditions. A vector coding for the inference score on a given test trial – derived by multiplying the correctness of the response (i.e., 0 or 1) with the confidence rating (i.e., 1 = guess, 2 = not sure, 3 = sure; see Supplemental Experimental Procedures)—was entered as a parametric regressor. Earlier regressors in the same general linear model captured effects attributable to changes in reaction time or overall performance (see Supplemental Experimental Procedures). Of note, the automatic serial orthogonalization procedure carried out by SPM8 results in shared variance among regressors being captured by earlier regressors. This procedure, therefore, allows one to ask in which brain regions neural activity during test trials tracks the development of successful transitivity choices supported by hierarchy knowledge, and cannot be explained by nonspecific effects—related to the contribution of alternative (e.g., procedural-based) mechanisms to overall performance, or changes in attention.

**Figure 2:**
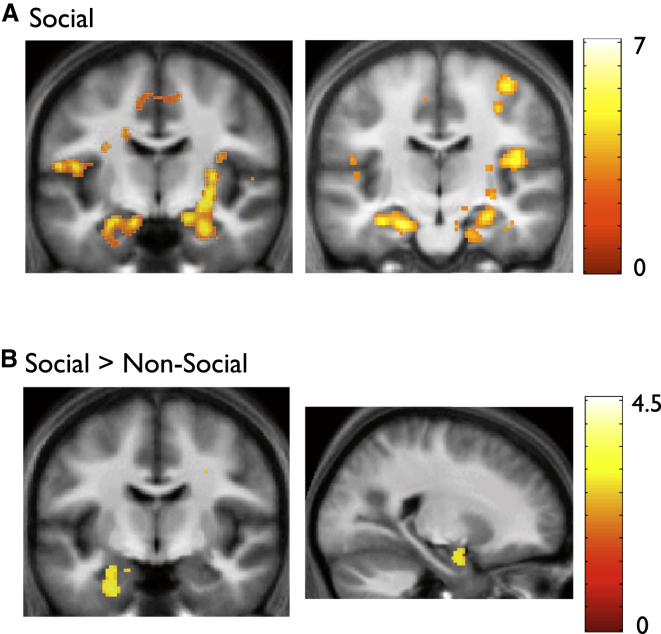
Learn Phase, fMRI Results: Neural Activity in the Amygdala and Anterior Hippocampus Selectively Tracks the Emergence of Knowledge about Social, but Not Nonsocial, Hierarchies (A) Activity in bilateral amygdala (left panel, coronal section) and bilateral anterior hippocampus (right panel, coronal section) shows a significant correlation with the inference score index in the social domain. Activations thresholded at p < 0.005 uncorrected for display purposes, and shown over average structural image of all participants but significant in amygdala and hippocampus at p < 0.001 uncorrected and p < 0.05 whole-brain FWE corrected at cluster level; colorbar (red-yellow) indicates increasing t values. See Table S1A for full list of activations. (B) Activity in left amygdala/anterior hippocampus (left panel, coronal section) and right amygdala (right panel, sagittal section) shows a significantly greater correlation with the inference score index in the social, as compared to the nonsocial, domain. Activations thresholded at p < 0.005 uncorrected for display purposes but significant in amygdala and hippocampus at p < 0.001 uncorrected and p < 0.05 SVC corrected; see Table S1B for full list of activations.

We first sought to identify brain regions where neural activity on a given test trial specifically tracked the development of knowledge about a social hierarchy, by using our trial-by-trial measure of transitivity performance—the inference score index - as leverage with which to interrogate the fMRI data. Strikingly, we found that neural activity within the amygdala and anterior hippocampus, as well as posterior hippocampus, and ventromedial prefrontal cortex (vMPFC), showed a significant correlation with the inference score index in the social domain (Figure 2A; Table S1A). Moreover, we found that the correlation between neural activity in the amygdala/anterior hippocampus and the inference score was specific to the social domain: no such correlation was observed in these regions even at liberal statistical thresholds (i.e., p < 0.01 uncorrected) in the nonsocial domain. Further, we observed that neural activity in these areas—in a cluster that included the left anterior hippocampus/amygdala, as well as right amygdala—showed a significantly greater correlation with the inference score in the social domain, as compared to the nonsocial domain (Figure 2B; Table S1B).

**Figure 3:**
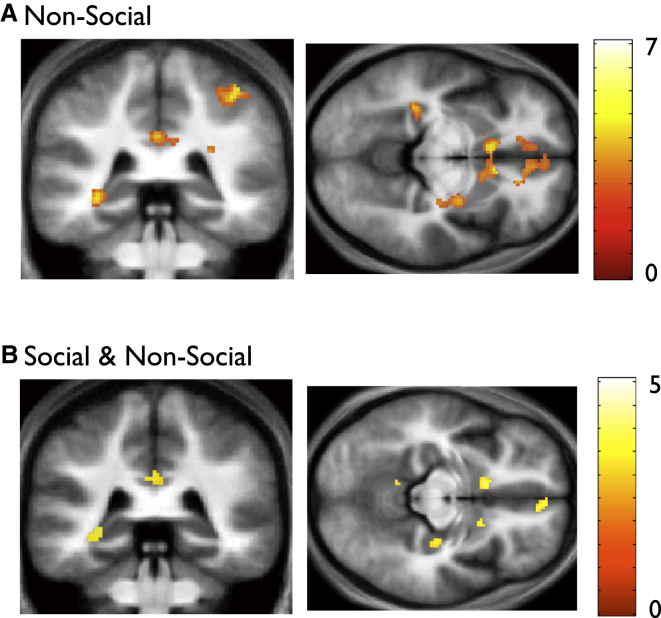
Learn Phase, fMRI Results: Neural Activity in the Posterior Hippocampus, and vMPFC, Tracks the Emergence of Hierarchical Knowledge in a Domain-General Fashion (A) Activity in left posterior hippocampus (left panel: coronal section, right panel: axial section) shows a significant correlation with the inference score index in the nonsocial domain. Activations thresholded at p < 0.005 uncorrected for display purposes, but significant in hippocampus and vMPFC at p < 0.001 uncorrected and p < 0.05 SVC corrected. No correlation was observed in the amygdala even at liberal statistical thresholds (p < 0.01 uncorrected). See Table S2A for full list of activations. (B) Results of conjunction (null) analysis: activity in left posterior hippocampus (left panel: coronal section) and vMPFC (right panel: axial section) shows a significant correlation with inference score index in both social and nonsocial domains. Activations thresholded at p < 0.005 uncorrected for display purposes, but significant in hippocampus and vMPFC at p < 0.001 uncorrected and p < 0.05 SVC corrected. See Table S2B for full list of activations.

Interestingly, as was the case in the social domain, we did observe a significant correlation between neural activity and inference score in the posterior hippocampus, and vMPFC, in the nonsocial domain (Figure 3A; Table S2A)—a finding that points toward a domain-general role for these regions, and which we further characterize in a subsequent (i.e., conjunction) analysis (see later and Table S2B). No brain regions exhibited a correlation that was significantly greater in the nonsocial, as compared to the social, domain (Table S2C).

Our evidence, in pointing toward a specific link between neural activity in the amygdala/anterior hippocampus and the development of knowledge about social hierarchies, is highly consistent with previous evidence suggesting that the amygdala plays an important role in emotional memory (McGaugh, 2004; Phelps and LeDoux, 2005; Somerville et al., 2006), through anatomical and functional interactions with the anterior hippocampus (Fanselow and Dong, 2010; Phelps, 2004). Importantly, the current findings relate closely to the development of social hierarchical knowledge and are not easily accounted for by less specific effects. First, we used a parametric approach—the fMRI results presented reflect a tight coupling between neural activity and participant-specific trial-by-trial regressors indexing hierarchical knowledge attained at a given time point during the Learn phase. As such, the findings reported from these parametric analyses cannot be explained by mere perceptual differences between the stimuli used in social and nonsocial domains (i.e., faces versus galaxies)—an account that would have had traction had we used a conventional subtractive strategy (i.e., social minus nonsocial).

Second, the robust correlation between neural activity in the amygdala/anterior hippocampus and participants’ performance in the social domain was restricted to test trials where performance depended on knowledge of the hierarchy—and not observed during training trials where a rote memorization strategy was sufficient (i.e., simply memorizing the correct item in a given training pair; see Supplemental Analysis 1 and Table S3A). Furthermore, the link between amygdala/anterior hippocampus activity and performance was found to be significantly greater during test trials, as compared to training trials, when we directly compared these two types of trials in an additional analysis where performance was captured solely by the correctness of participants’ choices (i.e., without inclusion of confidence ratings: see Supplemental Analysis 2 and Figure S1).

Finally, we examined the possibility that the observed correlation between neural activity in the amygdala/anterior hippocampus and social transitivity performance might have arisen due to the specific measure of test trial performance used (i.e., the inference score index)—and in particular the inclusion of participants’ confidence ratings. To examine this issue, we conducted a further analysis where test trial performance was captured solely by the binary choice data (i.e., as in Supplemental Analysis 2). Additionally, the time periods during which participants made their choices and rated their confidence were modeled separately in the general linear model (see Supplemental Analysis 3 and Figure S1). These data provide evidence that the correlation between neural activity in the amygdala/anterior hippocampus and transitivity performance is robust to the exclusion of the confidence data from the analysis—and relates specifically to successful choice during test trials, rather than participants’ metacognitive report about subjective confidence in their choice.

#### Neural Activity in the Posterior Hippocampus Tracks the Emergence of Knowledge about Hierarchies in a Domain-General Fashion

Given our evidence implicating the amygdala and anterior hippocampus selectively in knowledge about social hierarchies, we next sought to identify brain regions whose activity during test trials showed a significant correlation with the inference score index, in *both* social and nonsocial domains. To achieve this, we conducted a conjunction “null” analysis, which can be considered a conservative procedure for ensuring that each individual contrast is individually significant at a predefined threshold (i.e., p < 0.001 uncorrected for multiple comparisons; see Supplemental Experimental Procedures). Strikingly, we observed that neural activity in the posterior hippocampus, and the vMPFC, paralleled the emergence of knowledge about both social and nonsocial hierarchies (Figure 3B and Table S2B). These findings dovetail with accounts that the hippocampus, together with the vMPFC, plays a domain-general role during the emergence and application of relational knowledge (Cohen and Eichenbaum, 1993; Eichenbaum, 2004; Kumaran et al., 2009)—and accord with the observation that patients with damage to the vMPFC show a specific impairment in performing transitive inferences (Koscik and Tranel, 2012).

### Structural Neuroimaging Data: Voxel-Based Morphometry Analyses

#### Amygdala Gray Matter Volume Correlates Selectively with Transitivity Performance in Social Domain

Motivated by these results implicating the amygdala in the emergence of knowledge about social hierarchies and previous work linking variations in amygdala gray matter (GM) volume to interindividual differences in social network size in humans (Bickart et al., 2011; Kanai et al., 2012) and nonhuman primates (Barton and Aggleton, 2000; Sallet et al., 2011), we next performed a voxel-based morphometry (VBM) analysis (see Supplemental Experimental Procedures). Notably, the differing nature of the functional and structural analyses performed (i.e., within-subjects versus between-subjects, respectively) mean that the results so obtained provide independent lines of evidence concerning the neural substrates supporting knowledge about social hierarchies (see Supplemental Results).

**Figure 4:**
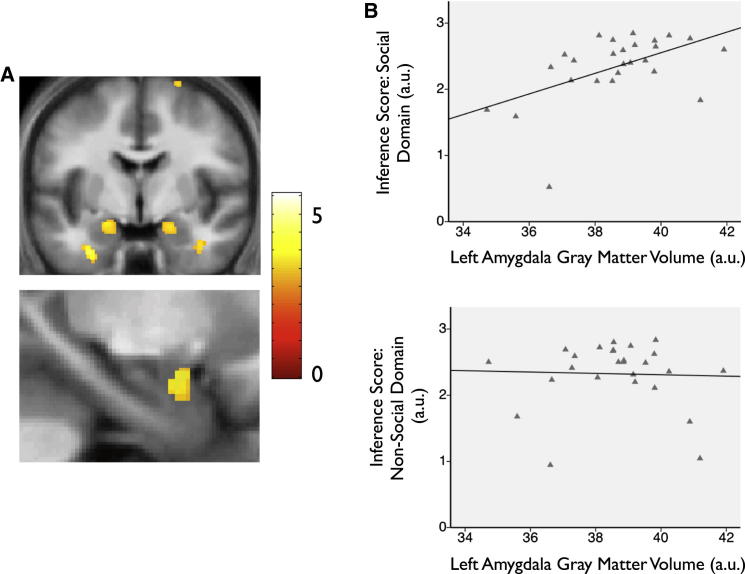
Learn Phase, VBM Results: Amygdala Gray Matter Volume Correlates Selectively with Transitivity Performance in Social Domain (A) Results of whole-brain analysis. Upper panel: gray matter volume in bilateral amygdala shows a significant between-subjects correlation with test trial performance in social domain, indexed by the inference score (averaged across experimental phase). Lower panel: close-up illustrating effect in left amygdala. Display threshold is p < 0.005 uncorrected but effects in bilateral amygdala are significant at p < 0.001 uncorrected, and p < 0.05 SVC corrected. Statistical maps displayed over the average structural image of participants. See Table S4A for full list of activations. (B) Results of ROI analysis. Upper panel: scatterplot illustrating significant correlation between left amygdala gray matter volume (averaged across anatomical ROI—see Supplemental Experimental Procedures) and intersubject differences in test trial performance, indexed by inference score, in social domain (left amygdala: r = 0.52 p = 0.003, also significant in right amygdala: r = 0.51 p = 0.004). This correlation remained significant in an analysis where the poorest performing participant was excluded, and hippocampal gray matter volume, performance on social training trials, and nonsocial inference score were partialled out (left and right amygdala, both ps < 0.01). Lower panel: scatterplot showing absence of a significant correlation between left amygdala GM and test trial performance in nonsocial domain (p > 0.1); see Supplemental Results for details.

We first carried out a whole-brain voxel-wise analysis to examine the relationship between GM volume and behavioral performance during the Learn phase. While participants achieved near-perfect knowledge of both hierarchies by the end of the experiment, individuals varied in their transitivity performance during the Learn phase. We observed that the variability in participants’ performance during test trials in the social domain, indexed by their inference score averaged across the whole experimental phase, was significantly predicted by interindividual variations in GM volume in the bilateral amygdala, and in no other brain regions (Figure 4A and Table S4A). Further, the correlation between GM volume in the amygdala and test trial performance was found to be significantly greater in the social, as compared to the nonsocial domain (Table S4B). No above threshold correlations were observed in the nonsocial domain (Table S4C).

We confirmed the robustness and specificity of the link between interindividual differences in the structure of the amygdala and social test trial (c.f. training trial) performance in two ways: first, by verifying that this correlation remained robust when test trial performance was captured solely by the binary choice data (i.e., with the confidence ratings excluded)—and second, in a region of interest (ROI) analysis in which GM volume was averaged across an anatomically defined mask (see Supplemental Results and Figure 4B). Notably, the observed correlations were highly specific to social transitivity judgments: no correlation was observed in relation to training trials where hierarchy knowledge was not required and a memorization strategy sufficient (p > 0.1; see Supplemental Results).

The results from the Learn phase provide converging evidence implicating the amygdala in the emergence of knowledge about social hierarchies. Taken together, our functional and structural findings point toward the conclusion that the amygdala, together with the hippocampus, participates in the representation of knowledge about social hierarchies—an account which draws upon the influential “memory storage” view of amygdala function (Phelps and LeDoux, 2005). Specifically, our fMRI results, in revealing a tight link between neural activity and performance during test trials, where no feedback was provided, suggests that the amygdala locally sustains neural representations of social hierarchies, rather than acting to facilitate their formation elsewhere (McGaugh, 2004). Furthermore, our VBM results—in showing that amygdala GM volume correlates with behavioral performance during social test trials—argue against a scenario in which the amygdala only provides a downstream signal that is triggered by the retrieval of hierarchy representations sustained elsewhere (e.g., in the hippocampus) and rather suggest that the amygdala itself contributes to the representation of knowledge about social hierarchies.

### Phase 2: Invest

In the next section of the fMRI experiment, we set out to probe participants’ recently established representations of the hierarchy and examine how rank information is coded in the brain. In particular, we wished to ask whether the amygdala might express a linear signal selectively coding for the rank of the individual person presented, when this information was motivationally relevant to behavior.

**Figure 5:**
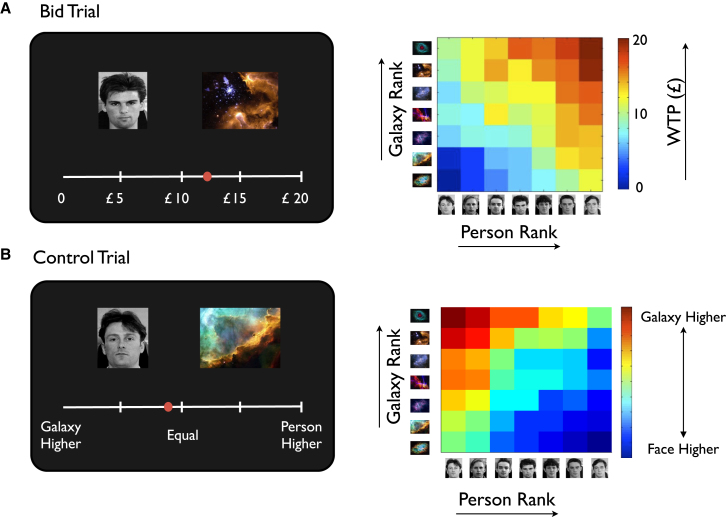
Invest Phase: Experimental Task and Behavioral Data (A) Bid trials: example trial (left) and behavioral data from a typical participant (right). Left panel: participants were required to state the maximum amount of money they would be willing to pay (i.e., WTP) to buy shares in potential projects on offer, denoted by the combination of a particular person and a particular galaxy (P4 and G2 shown), by positioning a cursor on a horizontal scale (a bid of £12.00 shown). The actual worth of the project was directly dependent on the rank of person and galaxy presented, and one trial was randomly selected to be played out at the end of the experiment as a real money transaction using the BDM mechanism (see Supplemental Experimental Procedures). Right panel shows a color coded heatmap of the prices a typical participant was willing to pay (i.e., WTP) for each of the 49 projects (i.e., all combinations of 7 galaxies and people in the hierarchy) on offer (x axis, person rank; y axis, galaxy rank; hot colors indicate higher WTP [from a minimum of £0 to a maximum of £20]). (B) Control trials: example trial (left) and behavioral data from a typical participant (right). Left panel: participants were required to determine which of the two items, person or galaxy (P7 G6 shown), was relatively higher in rank, and by how much: by positioning a cursor on a horizontal scale (response indicates that galaxy was deemed to be slightly higher in rank). As in bid trials, one trial was randomly selected at the end of the experiment and participants were rewarded according to the accuracy of their response. Right panel shows a color coded heatmap of the responses of a typical participant, for each of the 49 possible combinations of galaxies (x axis, person rank; y axis, galaxy rank). Hot colors indicate that the galaxy was the higher ranking of the two items.

During this phase of the experiment, participants viewed person-galaxy combinations and were required to complete two types of trials: bid and control trials (see Figure 5 and Supplemental Experimental Procedures). Importantly, person rank and galaxy rank were orthogonalized by experimental design—all 49 person-galaxy combinations were presented over trials—enabling us to characterize the relationship between neural activity and rank, separately for each stimulus type.

During bid trials, participants decided how much they would be willing to pay (i.e., WTP) for shares in potential projects on offer, based on their evaluation of the worth of individual people and galaxies (Figure 5A and Supplemental Experimental Procedures). Participants were instructed that the actual worth of projects was directly dependent on the rank of the items presented along the relevant dimension (i.e., person: power/galaxy: precious mineral content), and that one project would be selected at the end of the experiment to be played out as a financial transaction using the Becker-DeGroot-Marshak (BDM) mechanism (see Supplemental Experimental Procedures). Higher ranking items, therefore, were of greater motivational significance during bid trials—the expected monetary return of a given project depended on the rank of the items presented—with projects involving high ranking items (e.g., P1 G1) being inherently more valuable.

While control trials closely matched bid trials in terms of trial presentation and cognitive demands (e.g., use of knowledge about hierarchies), here participants were required to determine which item was higher in its respective “pecking order” and by how much (Figure 5B and Supplemental Experimental Procedures). In contrast to bid trials, therefore, higher ranking items were *not* of greater motivational significance than items occupying a lower rank position—as such control trials involving the presentation of highly ranked items (e.g., P1 G1) were not inherently more valuable in monetary terms to participants (c.f. trials involving lowly ranked items: e.g., P7 G7)—renumeration was based purely according to accuracy of cursor position on a randomly chosen trial.

### Behavioral Data

To characterize the influence of the rank of items presented on participants’ prices (i.e., WTP) during bid trials, and responses in the control task (i.e., indexed by position of the cursor on the scale), we performed a linear regression analysis (see Supplemental Experimental Procedures). This confirmed that participants afforded equal weight to both person and galaxy rank in their determination of the WTP of a given project, at a value that was close to optimal based on the instructions they had received (increase in WTP for each unit increase in person rank: £1.52 (SD 0.20), galaxy rank £1.55 (SD 0.29); no significant difference p > 0.1; optimal weighting = £1.67 per unit increase in person/galaxy rank; Figure 5A). Further, the influence of person and galaxy rank was not limited to the extremes of the hierarchy (i.e., highest or lowest ranked items) but was close to linear in fashion across the hierarchy (p < 0.001, r > 0.9). Similar findings were observed during control trials: equal weight was given to both person and galaxy rank (change in position of cursor on *x* axis for each unit change in person rank: 39.6 (SD 4.4); galaxy rank 40.7 (SD 7.0); no significant difference p > 0.1; optimal value = 41.5; Figure 5B). The influence of rank was also close to linear across the hierarchy (p < 0.001, r > 0.9), for both people and galaxies.

Further, participants rated the dimension of social rank as being subjectively realistic in the postexperimental debriefing session (mean 7.1 [out of 10], SD 2.9), attesting to the effectiveness of our experimental manipulation (see Supplemental Experimental Procedures). Participants were also asked to rate the face stimuli according to trustworthiness and attractiveness (i.e., in the debriefing session): while no significant correlation was observed between rank and these parameters (ps > 0.1), there was a significant correlation between ratings of trustworthiness and attractiveness in line with previous data (r = 0.44, p < 0.001; Todorov et al., 2008).

### Functional Neuroimaging Data (fMRI)

Given behavioral evidence that participants had deployed knowledge about both social and nonsocial hierarchies to inform their behavior in near-optimal fashion, we next turned to the fMRI data. We first set up a parametric model to identify brain regions whose activation pattern exhibited a significant linear correlation with the maximum amount of money participants were willing to pay for shares in a project during bid trials (i.e., WTP), with reaction time included in the model as a covariate of no interest (parametric model 1: see Supplemental Experimental Procedures).

**Figure 6:**
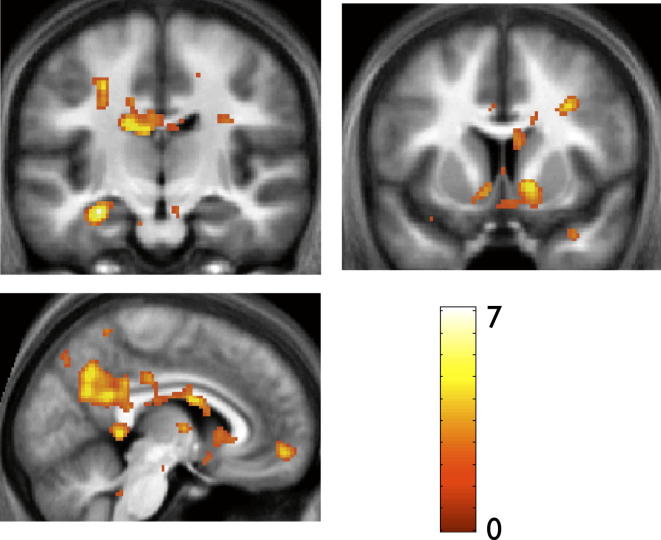
Invest Phase, fMRI Results: Brain Regions Whose Activity Showed a Significant Correlation with the Price Participants Were Willing to Pay (i.e., WTP) during Bid Trials Neural activity in the body of the left hippocampus (top panel, coronal section), nucleus accumbens (right panel, coronal section), posterior cingulate, and ventromedial prefrontal cortex (bottom panel, sagittal section) shows a significant correlation with participants’ stated prices (i.e., WTP) during bid trials. Activations thresholded at p < 0.005 uncorrected for display purposes but significant in hippocampus/nucleus accumbens/posterior cingulate cortex at p < 0.001 uncorrected and p < 0.05 whole-brain FWE corrected at cluster level—and in vMPFC at p < 0.001 uncorrected and p < 0.05 SVC corrected. See Table S5A for full list of activations.

We found that neural activity in the hippocampus and vMPFC showed a significant correlation with participants’ WTP (Figure 6 and Table S5A), consistent with previous work suggesting that the vMPFC encodes decision value during economic transactions through the integration of both social and nonsocial sources of value information (Rangel et al., 2008; Rushworth et al., 2011). Further, these findings provide support for perspectives proposing that the hippocampus and vMPFC may jointly contribute to goal-directed decision making, with the former neural structure housing recently acquired representations of the task structure which are passed to the latter for integration into choice behavior (Roy et al., 2012).

**Figure 7:**
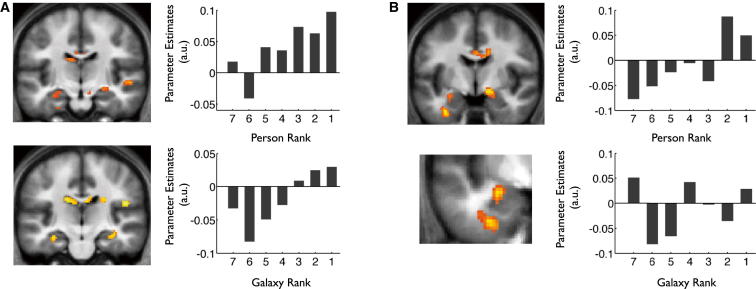
Invest Phase, fMRI Results: Evidence of Selective Person Rank Coding in Amygdala, and Domain-General Coding of Rank in Hippocampus, during Bid Trials (A) Domain-general coding of rank in hippocampus during bid trials. Left panels: results of whole-brain voxelwise analysis—neural activity in body of hippocampus shows significant correlation with person rank (top panel) and galaxy rank (bottom panel) during bid trials. Coronal sections show activations in hippocampal body. Activations thresholded at p < 0.005 uncorrected for display purposes, but significant at p < 0.001 uncorrected and p < 0.05 SVC corrected (see Tables S6A and S6B for full list of activations). Right panels: parameter estimates averaged across left hippocampal ROI defined based on an orthogonal selection contrast, as a function of person rank (top panel) and galaxy rank (bottom panel) (see Supplemental Results and Supplemental Experimental Procedures). Note these plots were derived from an “illustrative” model which included separate regressors for person and galaxy rank. Statistical inference, however, was based strictly on the parametric model. (B) Selective coding of person rank in amygdala during bid trials. Left panels: results of whole-brain voxel-wise analysis: activity in bilateral amygdala shows a significant correlation with person rank during bid trials: Top panel: coronal section, taken at level of peak in right amygdala. Bottom panel: close-up of activation in left amygdala at level of peak voxel. Activations are thresholded at p < 0.005 uncorrected for display purposes but are significant in amygdala at p < 0.05 SVC corrected (see Table S6A for full list of activations). Right panels illustrate parameter estimates averaged across left amygdala ROI defined based on an orthogonal selection contrast, as a function of person rank (above), and galaxy rank (below) (see Supplemental Results and Experimental Procedures). Significant linear correlation between neural activity in left amygdala and person, but not galaxy, rank evident.

We next sought to characterize the pattern of neural signals coding for rank information. To achieve this, we set up a parametric model in which the linear and quadratic effects of person and galaxy rank were modeled by separate regressors, with response time included as an additional regressor to control for nonspecific effects (fMRI parametric model 2; see Supplemental Experimental Procedures). While neural activity in the hippocampus, and vMPFC, showed a significant linear correlation with both person and galaxy rank during bid trials, the correlation in the amygdala was specific to person rank (Figures 7A and 7B; Table S6A). Indeed, no significant correlation was present between rank and amygdala activity in the nonsocial domain even at liberal statistical thresholds (i.e., p < 0.01 uncorrected; Table S6B). Further, equivalent findings were observed in an analysis where we included participant-specific ratings of attractiveness and trustworthiness obtained from the postexperimental debriefing session as regressors in the general linear model (see Supplemental Experimental Procedures). As such, our findings provide evidence that the profile of neural activity in the amygdala during bid trials specifically codes person rank, and cannot be accounted for along the lines of differences in perceived attractiveness or trustworthiness.

We next explored the specificity of the link between amygdala activity and person rank, and the effects of task context (i.e., bid versus control trials), by performing an ROI analysis (see Supplemental Experimental Procedures and Supplemental Results for full details of repeated-measures ANOVA). We observed that activity within an ROI within the left amygdala, which was functionally defined based on an orthogonal selection contrast (see Supplemental Experimental Procedures and Table S5B), showed a significantly greater correlation with person, as compared to galaxy, rank (t(24) = 2.3, two-tailed p = 0.03) during bid trials, an effect that was not present in a functionally defined region of the hippocampus (p > 0.1; see Supplemental Results). Moreover, person rank coding in the amygdala was observed to be significantly stronger during bid trials - where rank information was of direct motivational relevance—as compared to control trials (t(24) = 2.2, two-tailed p = 0.04; Figure S2 and Supplemental Results). Finally, we also found evidence linking the strength of the neural signal coding for person rank in the amygdala to behavior, with more robust coding in a given participant associated with greater influence of person rank on their WTP (r = 0.41, p = 0.02; see Supplemental Results).

In summary, the findings from the Invest phase suggest that the amygdala selectively expressed a signal coding for person rank during bid trials, where highly ranked individuals carried greater worth, and provide evidence of the behavioral significance of this signal. In contrast, our results indicate that the hippocampus plays a domain-general role in coding the rank of items in both social and nonsocial hierarchies. Our data relate closely to empirical evidence which demonstrates that the amygdala plays a role in representing the value of appetitive and aversive stimuli in the environment, in a fashion that is shaped by task context and motivational relevance, and can be closely linked to behavior (Balleine and Killcross, 2006; Baxter and Murray, 2002; Davis et al., 2010; Morrison and Salzman, 2010; Phelps and LeDoux, 2005). As such, our observation that neural activity within the amygdala tracks person rank during bid trials, as well as the finding that the robustness of amygdala coding of person rank across participants correlates with behavior, is highly consistent with previous work demonstrating that neural activity within the amygdala tracks stimulus-value associations, and can be tightly linked to behavioral output (e.g., Morrison and Salzman, 2010). Furthermore, the finding that amygdala coding of person rank was selective to bid trials and not observed in control trials where higher ranking individuals did not inherently signal higher monetary return, accords well with the notion that stimulus-value coding in the amygdala is exquisitely and rapidly sensitive to task context (e.g., Baxter and Murray, 2002).

However, it is important to note one important point of divergence between our data and domain-general accounts of value coding in the amygdala (e.g., Baxter and Murray, 2002; Morrison and Salzman, 2010): in our experiment, the amygdala was found to selectively code the worth of individuals based on their position in a social hierarchy, a finding which dovetails with the social-specific recruitment of the amygdala observed during the emergence of knowledge about hierarchies in the Learn phase. Importantly, this result cannot be explained by differences in terms of behavior: participants’ weighted person and galaxy rank equivalently during the decision process, with rank information influencing their WTP in a linear fashion in both domains.

One reason for the apparent discrepancy between our results and domain-general accounts of amygdala function is that value computation in our experiment was necessarily based on relational knowledge of a hierarchy (Cohen and Eichenbaum, 1993)—a qualitatively different experimental setting from the simpler forms of associative learning studied previously (Baxter and Murray, 2002; Davis et al., 2010; Morrison and Salzman, 2010). Alternatively, our findings may reflect a broader role for the amygdala in preferentially coding the value of social (c.f. nonsocial) stimuli during decision making (i.e., “decision values”; Rangel et al., 2008)—a hypothesis that merits scrutiny given the paucity of studies that have examined this question. Notably, previous work that has examined the role of the amygdala in coding stimulus values have typically explored this question separately in social (Davis et al., 2010) and nonsocial domains (Morrison and Salzman, 2010). As such, the few studies that have directly compared value computation in social and nonsocial domains have done so in a quite different experimental context—involving the processing of rewarding outcomes (i.e., “experienced value”) such as attractive faces (social) and money (nonsocial) (Lin et al., 2011; Smith et al., 2010). In the future, it will be of interest to ask whether our finding, that the amygdala plays a selective role in coding decision values in the social domain based on hierarchical knowledge, generalizes to a wider range of experimental scenarios.

### The Amygdala and Knowledge about Social Hierarchies

Taken together, the present study provides converging evidence, obtained using a combination of structural and functional neuroimaging techniques, which specifically implicates the amygdala in the emergence of knowledge about a social hierarchy through experience. Our findings further demonstrate that neural activity in the amygdala selectively discloses the worth of other individuals based on their rank, a signal that could potentially be useful in guiding the selection of advantageous coalition partners (Cheney and Seyfarth, 1990; Tomasello and Call, 1997).

Further investigation, however, is required to delineate the full range of conditions under which the amygdala is recruited. Our paradigm was motivated by the emphasis placed on the dimension of power/dominance in organizing social hierarchies in human and nonhuman primates (Cheney and Seyfarth, 1990; Cummins, 2000; Magee and Galinsky, 2008). Nevertheless, it is worth noting that social hierarchies are often viewed to extend beyond the dimension of power—as such, they have been more broadly construed as denoting the rank order of individuals with respect to *any* valued social dimension (Magee and Galinsky, 2008). It would be potentially illuminating, therefore, to ask whether the amygdala might be similarly recruited when participants acquired knowledge of a social hierarchy where individuals were ranked according to another valued social dimension—namely trustworthiness—an experiment that would have particular relevance given the importance of this dimension to the evaluation of unfamiliar faces based on perceptual information (see discussion later; Adolphs et al., 1998; Todorov et al., 2008; Winston et al., 2002). Furthermore, one could also examine the relationship between the nature of stimulus used to depict different individuals in the hierarchy, and the recruitment of the amygdala. While our experiment was guided by the pivotal role attributed to visual face processing in the learning and expression of knowledge about social hierarchies (Byrne and Bates, 2010; Cheney and Seyfarth, 1990; Deaner et al., 2005), one could conceive of a scenario in which symbolic stimuli (e.g., person names) were used, instead of face images. Though such an experimental design would likely not eliminate the operation of visual face processing—participants would likely conjure up images of familiar people to associate with each name—future investigation along these lines may help to further characterize the contribution of the amygdala to supporting knowledge about social hierarchies.

In contrast to our study, previous work has tended to explore how the dominance of individuals that have never previously been encountered is judged based on perceptual cues (Karafin et al., 2004; Todorov et al., 2008; Marsh et al., 2009; also see: Thomsen et al., 2011)—rather than information about their rank in the hierarchy acquired through experience. One avenue of research has examined how unfamiliar individuals are rapidly evaluated based on visual information present in face features, according to two principal dimensions of valence/trustworthiness and power/dominance (Todorov et al., 2008; Whalen, 1998). While substantial data suggests that the amygdala codes the trustworthiness of an unfamiliar face based on perceptual features (Adolphs et al., 1998; Winston et al., 2002), evidence concerning its role in signaling dominance has been lacking (Todorov et al., 2011). Our study, by revealing the existence of a robust signal coding for the rank of an individual based on knowledge of a social hierarchy (c.f. perceptual features), provide support for the hypothesis that the amygdala may be engaged in dominance evaluation, when this dimension is of motivational importance (Todorov et al., 2008).

A largely separate line of work has investigated how more general cues of status, such as body posture and attire, influence behavior (Galinsky et al., 2003; Keltner et al., 2003), dominance judgments (Karafin et al., 2004; Mah et al., 2004), and neural processing (Marsh et al., 2009; Zink et al., 2008). These previous studies have shown that activity in the prefrontal cortex, and in certain conditions the amygdala, is upregulated when participants view high status individuals, where information about status is conveyed through their body posture (e.g., outward pose) (Marsh et al., 2009) or explicitly presented (i.e., star rating) (Zink et al., 2008). For instance, in a study by Marsh et al. (2009), increased activity in the ventrolateral prefrontal cortex was observed when participants viewed images of a high-status (c.f. low-status) individual, whose status was revealed by their physical appearance (e.g., body posture and gaze direction), rather than learned through experience as in our experiment. In the future, it will be important to integrate these different strands of research—in particular, it will be interesting to explore the neural mechanisms by which individuals integrate perceptual information (e.g., facial appearance, body posture), information gained through linguistic discourse with their peers, with knowledge about the social hierarchy of their group that has been acquired through experience, to make accurate judgments of the rank of others.

### The Hippocampus: Neural Representations of Social and Nonsocial Hierarchies

While previous work has implicated the hippocampus in the generation of transitive inferences (e.g., Dusek and Eichenbaum, 1997), there has been little direct evidence concerning its role in the emergence and representation of knowledge about linear hierarchies, despite the pervasive influence of these structures across a range of cognitive domains (Kemp and Tenenbaum, 2008). In contrast to previous studies (e.g., Moses et al., 2010), our experiment was specifically set up to examine how knowledge about hierarchies develops through experience and is represented at the neural level—through the incorporation of trial-by-trial behavioral indices in each experimental phase (e.g., inference score) that permitted investigation of the underlying neural mechanisms.

Our data point to the existence of a dissociation between the respective roles of the anterior and posterior regions of the hippocampus during the emergence of knowledge about hierarchies. As such, the anterior hippocampus, and the amygdala, were selectively recruited during the emergence of knowledge about a social hierarchy—a finding that sits comfortably with the massive bidirectional connectivity between these two regions, and their synergistic contribution to emotional memory (Fanselow and Dong, 2010). It is interesting to relate the current findings to those of a previous study (Kumaran and Maguire, 2005), which found that knowledge of one’s social network—another variety of social knowledge with relational qualities (i.e., capturing the relations that exist between different individuals)—was supported by neocortical regions including the medial prefrontal cortex and superior temporal sulcus, rather than structures within the medial temporal lobe. Critically, however, in this study participants’ knowledge of their social network was well established, having been acquired several months previously. While further work is required, these findings collectively suggest that the hippocampus may play a role during the initial emergence and representation of relational forms of social knowledge (Cohen and Eichenbaum, 1993)—but that this information is ultimately consolidated to the neocortex for long-term storage (Eichenbaum, 2004; McClelland et al., 1995).

In contrast to the social-specific recruitment of the anterior hippocampus observed during the emergence of knowledge about hierarchies during the Learn phase, the engagement of the posterior hippocampus was domain general in nature. Further, the hippocampal body was found to code the rank of individual items in a domain-general fashion during the Invest phase, providing compelling evidence that the linear structure of hierarchies is represented at the neural level. Together, these data suggest that the hippocampus supports domain-general representations of hierarchical knowledge and provide insights into how such information may be integrated into the computation of decision values, putatively in regions such as the vMPFC (Rangel et al., 2008; Roy et al., 2012; Rushworth et al., 2011). More generally, the present study adds to growing evidence that the hippocampus may play an important role in supporting neural representations that code for the overall structure of a set of related experiences (Eichenbaum, 2004; Kumaran et al., 2009; Shohamy and Wagner, 2008) and highlights the need for formal computational models that are able to marry such a function with its widely acknowledged role in episodic memory (Cohen and Eichenbaum, 1993; McClelland et al., 1995).

### Conclusions

Primates possess sophisticated knowledge of the rank relations that exist between fellow members of their social group (Byrne and Bates, 2010; Cheney and Seyfarth, 1990; Tomasello and Call, 1997), yet surprisingly little is understood about the underlying neural mechanisms. Our data offer concrete evidence that the amygdala forms part of the specialized neural machinery that operates during the emergence and expression of knowledge about social hierarchies and illuminates the distinct contribution of the hippocampus to the domain-general representation of hierarchies. More generally, the current study provides support for the viewpoint that the evolution of the primate amygdala may have been driven by the challenging cognitive demands of a rich and varied social life (Adolphs, 2003)—and underscores the importance of defining how other forms of social knowledge (c.f. social networks) may emerge from a rich tapestry of previous experiences.

## Experimental Procedures

Here, we provide an overview of the experimental tasks and the procedures used to analyze the fMRI data; full details are provided in the Supplemental Experimental Procedures.

### Participants

Twenty-six healthy, right-handed individuals who were currently undertaking or had completed a university degree, participated in this experiment (age range 19–31; 12 female). One of these participants failed to fully learn either person or galaxy hierarchies and was therefore excluded from the fMRI analyses. All participants gave informed written consent to participation in accordance with the local research ethics committee.

### Stimuli

Face pictures were obtained from a widely used database (Stirling database: http://pics.stir.ac.uk). Pictures of galaxies (source: various sites on the internet including http://hubblesite.org/gallery/album/nebula) were chosen to be distinct from one another. Prior to each scanning session, participants briefly performed a simple one-back task where they viewed each individual face and galaxy three times, in order to minimize stimulus novelty effects during scanning.

### Description of Tasks

Participants were instructed that they would be playing a simple science-fiction computer game, acting as an investor in the future. They were told they would first (“Learn” phase) need to learn about which individuals have more power within a fictitious space mining company and which galaxies have more precious mineral. In phase two (“Invest” phase), they were told that they would need to use knowledge acquired during phase one to decide how much they would be willing to pay for potential projects on offer—where a project constituted the combination of a particular person and a particular galaxy (i.e., as if the person would be heading up a mission to go to the galaxy to harvest minerals).

The Learn phase paradigm is grounded in classic implementations of the transitive inference task (Bryant and Trabasso, 1971), where dimensions such as length and weight were emphasized (c.f. mineral content in our study). In this phase, participants acquired knowledge about the seven-item person and galaxy hierarchies in parallel with blocks of training trials (i.e., six training pairs, presented in pseudorandom order: e.g., P1 versus P2, P2 versus P3, P3 versus P4, P4 versus P5, P5 versus P6, P6 versus P7; see Figure 1A for details) interleaved with blocks of test trials (i.e., six inference pairs, presented in pseudorandom order: e.g., P2 versus P4, P2 versus P5, P2 versus P6, P3 versus P5, P3 versus P6, P4 versus P6; see Figure 1B; for details, see Supplemental Experimental Procedures).

In the Invest phase, participants were required to use their knowledge about person and galaxy hierarchies to decide (1) how much in real monetary terms to pay for potential projects on offer (“bid” trials: see Figure 5A and Supplemental Experimental Procedures), by evaluating the potential worth of individual people and galaxies based on their rank, or (2) which item (i.e., person or galaxy) was more highly ranked, and by how much (“control” trials: see Figure 5B).

### fMRI Data Acquisition

T2 weighted gradient-echo planar images (EPI) with BOLD (blood oxygen level-dependent) contrast were acquired on a 3.0 tesla Siemens Allegra MRI scanner using a specialized sequence to acquire whole-brain coverage, while minimizing signal dropout in the medial temporal lobe and ventromedial prefrontal cortex. High-resolution (1 × 1 × 1 mm) T1-weighted structural MRI scan were also acquired for each participant after functional scanning.

### fMRI Data Analysis

Images were preprocessed and analyzed in a standard manner using the statistical parametric mapping software SPM8 (www.fil.ion.ucl.ac.uk/SPM). Details of the parametric models used are given below: see Supplemental Information for full details of procedures used for model specification, estimation, statistical inference, and ROI analyses.

#### Specification of First-Level Design Matrix: Phase 1 (Learn)

The following participant-specific trial-by-trial parametric regressors were included (in the order stated) in the first level design matrix relating to test trials (see Supplemental Information for specification of training trial, and other regressors also included in the model [e.g., movement parameters, etc.]):

(1) Trial-by-trial reaction time (RT); (2) probability_correct: following previous studies (e.g., Kumaran et al., 2009) trial-by-trial estimates of the probability of a correct response derived from learning curves were constructed separately for each of the six test pairs (e.g., P2 P4) using the state-space model (Smith et al., 2004); (3) inference score (range 0–3; see above).

All test trial types (i.e., six pairs: P2 versus P4, P2 versus P5, P2 versus P6, P3 versus P5, P3 versus P6, P4 versus P6) were modeled within these regressors, with one regressor for the person condition and one for the galaxy condition.

#### Specification of First-Level Design Matrix: Phase 2 (Invest)

We set up two different parametric models to detect brain regions whose activation pattern (1) exhibited a significant linear correlation with the maximum amount of money participants were willing to pay for shares in a project during bid trials (i.e., WTP) and (2) showed a significant linear correlation with the rank of person or galaxy in the hierarchy, during bid or control trials.

The following vectors were then included as parametric modulators in the design matrix (in order): fMRI parametric model one—(1) trial-by-trial reaction time (RT), (2) WTP: participants’ stated maximum price that they were willing to pay for the shares in the project; fMRI parametric model two—(1) trial-by-trial reaction time (RT), (2) galaxy rank, (3) person rank.

These parametric regressors were convolved with the HRF, leading to the height of the HRF for a given event being modulated accordingly. Thus, these regressors model BOLD signal changes that covary with specific behavioral indices of performance on a given trial (e.g., inference score during test trials in phase 1).

### Statistical Inference

We report results in a priori regions of interest—the hippocampus, amygdala and ventromedial prefrontal cortex—where activations are significant at p < 0.001 uncorrected for multiple comparisons and survive small volume correction (SVC) for multiple comparisons (at p < 0.05 corrected) using SPM8 (e.g., using anatomical masks for hippocampus and amygdala; see Supplemental Experimental Procedures for details). Activations in other brain regions were only considered significant if they were significant at a level of p < 0.001 uncorrected and additionally survived whole brain FWE correction at the cluster level (p < 0.05 corrected).
