# Supplementary material for: The Emergence and Representation of Knowledge about Social and Nonsocial Hierarchies
Source: Neuron. 2012 Nov 8;76(3):653–66. doi: 10.1016/j.neuron.2012.09.035 (PMC3580285; doi:10.1016/j.neuron.2012.09.035)
Supplement: Document S1. Figures S1 and S2, Tables S1–S6, and Supplemental Text [file mmc1.pdf]

Neuron, Volume *76*

Supplemental Information

The Emergence and Representation of Knowledge about Social and Nonsocial Hierarchies

Dharshan Kumaran, Hans Ludwig Melo, and Emrah Duzel1

INVENTORY OF SUPPLEMENTAL INFORMATION

Supplemental Results

-Rationale behind the Inference score and details of independent behavioral experiment validating this measure (Learn phase)

-Supplemental analyses demonstrating specificity of amygdala/anterior hippocampal activity to test trials (Learn phase), and results from analyses where test trial performance was indexed by binary choice data only.

- Supplemental VBM region-of-interest analyses (Learn phase)
- Supplemental Region-of-Interest (Invest phase)

Supplemental Figures and Legends (S1-2)

- Figure S1: correlation between amygdala/hippocampal activity is specific to test trials (Learn Phase), and robust to the exclusion of the confidence data from the analysis.
- Figure S2: Amygdala selectively codes person rank during bid trials (ROI analysis: Invest phase)

Supplemental Tables (S1-S6)

- Tables S1-S3: fMRI Results: Learn phase
- Table S4: VBM Results Learn phase
- Tables S5-S6: fMRI Results: Invest phase

Supplemental Experimental Procedures

-Complete description of experimental tasks, fMRI and VBM analytic techniques.

Supplemental References

-List of references cited in the supplemental information

Appendix

-Full details of the instructions given to participants (Learn, Invest phases)

SUPPLEMENTAL RESULTS

Learn Phase: Behavioral Data

Rationale behind use of inference score index during Learn Phase.

The overall goal of the Learn phase paradigm was to provide an experimental setting in which participants would gradually acquire robust knowledge of the linear hierarchy, use this to make successful transitive inferences during test trials, and afford a means by which could track this process at behavioral and neural levels. Importantly, our aim was to target one specific type of neural representation –a relational representation of the hierarchy (i.e. A>B>C…>F : e.g. Cohen and Eichenbaum, 1993; Dusek and Eichenbaum, 1997; Smith and Squire, 2005) – and thereby go beyond previous work in this field which has found it difficult to cleanly distinguish between the contribution of relational and procedural reinforcement-based mechanisms to successful transitive choices (Frank et al., 2003; Frank et al. 2005; Greene et al., 2006).

To achieve this, we set out to develop a behavioral index which would afford a block-by-block assessment of the level of hierarchy knowledge attained across the timecourse of learning, and thereby act as an online proxy for what is generally viewed as the "gold standard" test of relational hierarchy knowledge (e.g. Smith and Squire, 2005) – the post-scan test where participants were required to construct the rank order of items in the hierarchy. Our rationale for using confidence data, as well as binary choice data, to capture test trial performance was two fold: 1) to index gradations in first-order processes that support task performance (Fleming et al., 2012) 2) to specifically target the contribution of relational memory representations of the hierarchy to transitivity performance, over the potential influence of procedural reinforcement-based representations, drawing on previous applications of confidence data, and related procedures in nonhuman primates and rodents (e.g. using changes in response criteria e.g. (Fortin et al., 2004; Guderian et al., 2011), to characterize MTL-dependent memory processes (e.g. (Eichenbaum et al., 2007; Squire et al., 2007). In this way, we aimed to gain a fine-grained estimate of the strength of the underlying hierarchy representation across the Learn phase of the experiment.

Results of Independent Behavioral Study: Critically, we validated this novel measure of test trial performance in an independent behavioral study (see below) involving 27 healthy university students, none of whom took part in the fMRI study. In this way, we show that the inference score has objective explanatory value, over and beyond the binary choice data, in predicting the level of hierarchy knowledge attained by a given participant (i.e. as directly measured by the gold-standard test: Smith and Squire, 2005).

The design of this behavioral experiment was similar to that of the current fMRI experiment: involving interleaved training and test blocks, and the use of a 3 point confidence scale during test trials, though participants were only required to learn a galaxy hierarchy. In contrast, however, this behavioral study was tailored (e.g. longer 8-item hierarchy, fewer training blocks) to foster greater inter-individual variability in participants' relational knowledge of the hierarchy as demonstrated in the post-experimental hierarchy recall test. In this way, we were able to ask whether the inference score index provides a more robust measure of participants' knowledge of the hierarchy, as compared to the binary choice data (i.e. correctness of participants' responses). Indeed, this is what we observed - the inference score (averaged across the last block of learning) was found to show a significant correlation ($r=0.7$ $p<0.001$) with participants' knowledge of the linear structure of the items, an effect that remained robust when both the correctness of participants' responses during test trials, and training trials, were partialled out (i.e $r=0.4$ $p<0.05$).

To summarize, our data suggest that the inference score index both furnishes an online assessment of participants' capacity for successful inference, and provides a window into the level of relational hierarchy knowledge attained across the timecourse of learning.

Learn Phase: Functional Neuroimaging Data

The link between amygdala/anterior hippocampal activity and performance is specific to transitive judgements of social rank, and remains robust to the exclusion of the confidence data.

Supplemental Analysis 1: We carried out an analysis designed to identify brain regions whose activity during *training trials* showed a significant correlation with proficient performance, modelled by the probability_correct parametric regressor, over and above non-specific effects due to changes in reaction time (see Supplemental Experimental Procedures). In marked contrast to the robust activity observed in the amygdala/anterior hippocampus observed in relation to performance in social test trials – no brain regions showed a statistically significant correlation between performance in social training trials (Table S3A). Further, no significant differences were observed in this analysis when we directly compared social and non-social domains (Table S3B) – with no activity in the amygdala observed even at liberal thresholds (i.e. $p<0.01$ uncorrected).

Supplemental Analysis 2: We also conducted an analysis that was designed to identify brain regions whose activity showed a significantly greater correlation with performance during *test trials*, where knowledge of the hierarchy was required, as compared to training trials where a rote memorization strategy was sufficient. In the main analyses reported, test trial performance was captured by the inference score index, which was derived by combining the correctness of participants' response with their confidence rating, to provide specific leverage on the evolution of hierarchical knowledge (see above). This supplemental analysis was configured to facilitate a direct comparison between two trial types: as such training and test trial performance was modelled through a regressor based solely on the binary performance data, which was entered as a second parametric regressor against an earlier regressor capturing trial-by-trial reaction time - i.e. test trial confidence ratings were not included in the analysis.

In line with the results obtained from our principal analyses (i.e. where test trial performance was captured by the inference score index: Figure 2), activity in the

amygdala/anterior hippocampus, and in no other brain regions, showed a significantly greater correlation with successful performance during test trials, as compared to training trials ($p<0.001$ uncorrected and SVC $p<0.05$ corrected: Figure S1A). No above threshold activations were observed in the reverse comparison (i.e. training > test trials). This finding provides additional support for the assertion that our finding with respect to the amygdala is highly specific to the emergence of a capacity for successful transitive judgements of social rank, underpinned by knowledge about a social hierarchy, and does not reflect a more general correlation with proficient performance in the social domain.

Further, the specificity of our findings to test trials also argues against an alternative account based on changing novelty responses to the faces themselves. Indeed, novelty-related effects were additionally minimized by a conventional procedure (i.e. by pre-exposing participants to the stimuli prior to the start of the experiment: see Supplemental Experimental Procedures), and would in any case be expected to produce a decrease in neural activity (i.e. habituation) across the Learn phase, rather than the parallel rise of neural activity and behavioral (i.e. transitivity) performance as was observed. Finally, it is worth noting that our results cannot be explained by non-specific effects (e.g. scanner drift) over the experimental phase - a qualitatively similar pattern of findings was observed when such effects of time were included as regressors in the general linear model.

Supplemental Analysis 3: We examined the possibility that the observed correlation between neural activity in the amygdala/hippocampus and transitivity performance might have arisen due to the specific measure used to capture test trial performance (i.e. the inference score index), and in particular the incorporation of participants' confidence ratings in the analysis. In this analysis, test trial performance was indexed solely using the binary choice data. The time period during which participants' made their choice during test trials was modeled as a separate regressor, parametrically modulated by the correctness of their responses- trial-by-trial reaction time was also included as an earlier parametric regressor. Additionally, a separate regressor, coding for the time period during which participants reported their confidence, was included to model neural activity at the

time of metacognitive report. Consequently, these analyses were set up to identify neural activity at the time of choice that correlates with successful test trial performance.

Results from these additional fMRI analyses based on the binary choice data reveal a qualitatively similar pattern of findings to the principal analyses conducted with the inference score index (see Figure S1). Neural activity in the amygdala, and anterior hippocampus, showed a robust correlation with transitivity performance in the social domain ($p<0.05$ FWE corrected at cluster level). No significant correlation was observed in these regions in the non-social domain, even at liberal statistical thresholds (i.e. $p<0.01$ uncorrected) - rather, neural activity in posterior hippocampus and VMPFC was correlated with successful test trial performance in this context ($p<0.05$ SVC corrected). Furthermore, activity in the amygdala, and anterior hippocampus, showed a significantly greater correlation with transitivity performance in the social, as compared to the non-social, domain ($p<0.05$ SVC corrected). As such, neural activity in the amygdala was found to be selectively linked to successful transitivity performance in the social domain - in contrast to the significant correlation between neural activity in the hippocampus and vMPFC with transitivity performance observed in both social and non-social domains ($p<0.05$ SVC corrected).

As a further step, we also performed an analysis where participants' raw confidence ratings (i.e. regardless of the correctness of response) were also included in the general linear model, as a parametric modulator of the regressor encoding the time period of confidence judgments. Of note, this can be considered a conservative analysis given that raw confidence ratings inherently show a degree of correlation with the binary choice data (average correlation across participants and conditions ~ 0.4), thereby reducing the statistical power of detecting significant choice-related effects. Nevertheless, our findings demonstrate that the profile of findings was qualitatively similar to the findings reported above: as such, neural activity in the right amygdala (and anterior hippocampus) showed a robust correlation with the correctness of transitivity choices that was restricted to the social domain - and was significantly greater than in the non-social domain ($p<0.05$ SVC).

Taken together, the results from these additional analyses provide evidence that the correlation between neural activity in the amygdala/hippocampus and transitivity performance does not rely on the use of the inference score index, and is robust to the exclusion of the confidence data from the analysis. Further, these findings indicate that neural activity observed in the amygdala/hippocampus relates specifically to successful transitive choices during test trials, rather than subsequent metacognitive report. In support of this overall conclusion, no significant activity in the amygdala was observed even at liberal statistical thresholds ($p<0.01$ uncorrected for multiple comparisons), when neural activity at the time of metacognitive report, captured by the parametric regressor coding for participants' confidence judgments, was directly compared between social and non-social domains.

Learn Phase: Structural Neuroimaging Data

Voxel-Based Morphometry: Region-of-Interest (ROI) Analyses

We confirmed the robustness and specificity of the link between interindividual differences in the structure of the amygdala and social test trial performance in two ways: firstly, we performed an additional analysis where test trial performance was captured solely by the binary choice data (i.e. without inclusion of the confidence ratings). As in the principal analysis involving the inference score index, we observed a significant correlation in a whole brain voxel-wise analysis, and in a ROI analysis (see below), between amygdala GM volume and test trial performance in the social domain, that was significantly greater than that observed in the non-social domain (i.e. significant at $p<0.001$ uncorrected and $p<0.05$ SVC corrected).

Secondly, we performed an ROI analysis in which GM volume was averaged across an anatomical defined mask. As in the voxel-based analysis, we observed a significant correlation between amygdala GM volume and social (left amygdala: $r=0.52$ $p=0.003$, right amygdala: $r=0.51$ $p=0.004$)(Figure 4B top panel), but not non-social ($p>0.1$)(Figure 4B bottom panel), test trial performance. Moreover, no such correlation was observed with training trial performance in either domains ($p>0.1$), pointing towards an intimate link between increased GM volume in the amygdala and social hierarchical knowledge.

In contrast, no such correlation was found between hippocampal GM volume (both left, right $p>0.1$) and test trial performance in either social or non-social domains. Further, the correlation between amygdala GM volume and social test trial performance remained significant when the poorest performing participant was excluded (left amygdala: $p=0.004$, right amygdala $p=0.005$), and in a regression model where other factors -- specifically, age, sex, non-social inference score, training trial performance in social domain and left hippocampal GM volume-- were partialled out (both left and right amygdala: $p<0.01$). Of note, an analogous set of findings - with significant correlations between amygdala GM volume and social test trial performance (both amygdala $p<0.01$) - were observed in a supplemental analysis where performance was captured using only the binary choice data , rather than the inference score index.

We also conducted a median-split analysis, where we divided participants into two groups according to our behavioral index of test trial performance (i.e. averaged inference score across the Learn phase), and asked whether amygdala GM volume was significantly greater in "good" test trial performers, as compared to "poor" performers. A significant effect was found in this analysis in the left amygdala (poor group left amygdala GM volume: 38.0 (SD 1.4), good group GM volume 39.2 (SD 1.8)), with a trend observed in the right amygdala (left amygdala: $t_{12}=2.4$ $p<0.05$, right amygdala: $t_{12}=1.8$, $p=0.09$).

Invest Phase: Neuroimaging Data

Region-of-Interest Analysis: Amygdala activity shows a selective linear correlation with person rank during bid trials, the robustness of which influences participants' behavior.

We next explored the specificity of the link between amygdala activity and person rank, and the effects of task context (i.e. bid vs control trials), by performing a ROI analysis. Regions of interest in the left amygdala, and left hippocampus (a comparison region) were functionally defined based on an orthogonal selection contrast (see Supplemental Experimental Procedures and S5B for full list of activations observed in this contrast).

Importantly, our analysis fulfils the criteria outlined by Kriegeskorte et al (2009): the definition of these ROI is *unbiased*, and therefore statistically independent, with respect to the contrasts relevant to addressing our two experimental questions of interest concerning the amygdala. Specifically, the main effect contrast used to define the ROIs is orthogonal to the relevant contrasts of interest (i.e. their matrix dot product equals zero). Further, all other parameters were balanced across the two tasks and stimulus types (i.e. person, galaxy), for example the number of experimental trials, preventing other sources of bias entering into the analysis.

Parameter estimates, averaged across these ROIs, were entered into a repeated measures ANOVA with factors: region (left amygdala, left hippocampus), task (Bid, Control) and hierarchy type (Person, Galaxy). A significant three-way interaction, region x task x hierarchy type, was observed in this analysis: $F(1,24)=8.2$ $p=0.009$ (no significant main effects, $p>0.1$). Further analyses showed that this effect was driven by selective coding of person rank in the amygdala during bid trials (Figure S2): as such, there was a significant interaction between task and hierarchy type in the left amygdala ($F1,24=4.1$, $p=0.05$), but not in the hippocampus ($p>0.1$). Paired t-tests confirmed that there was a significantly stronger link between neural activity and person, as compared to galaxy, rank in this region of the left amygdala during bid trials ($t(24)=2.3$, $p=0.03$ 2-tailed). In contrast, no such effect was observed in the hippocampus (person > galaxy: $p>0.1$), which exhibited significant coding of both person rank and galaxy rank during bid trials (both $ps<0.05$). Finally, there was a significantly stronger link between neural activity and person rank in the left amygdala during bid trials, as compared to control trials ($t(24)=2.2$, $p=0.04$ 2-tailed), an effect that was not present in the hippocampus $p>0.1$.

Interestingly, we also found that interindividual differences in the robustness of person rank coding in the amygdala correlated with participants' behavior: specifically, the influence of person rank on participants' WTP was significantly predicted by the strength of the linear correlation (i.e. parameter estimate) between neural activity in the amygdala and person ($r=0.41$, 1-tailed t-test $p=0.02$) rank. No such correlation was observed

between the influence of galaxy rank on participants' WTP and the strength of galaxy rank coding in the amygdala ($r=0.02$, $p>0.1$).

Supplemental Figures and Legends.

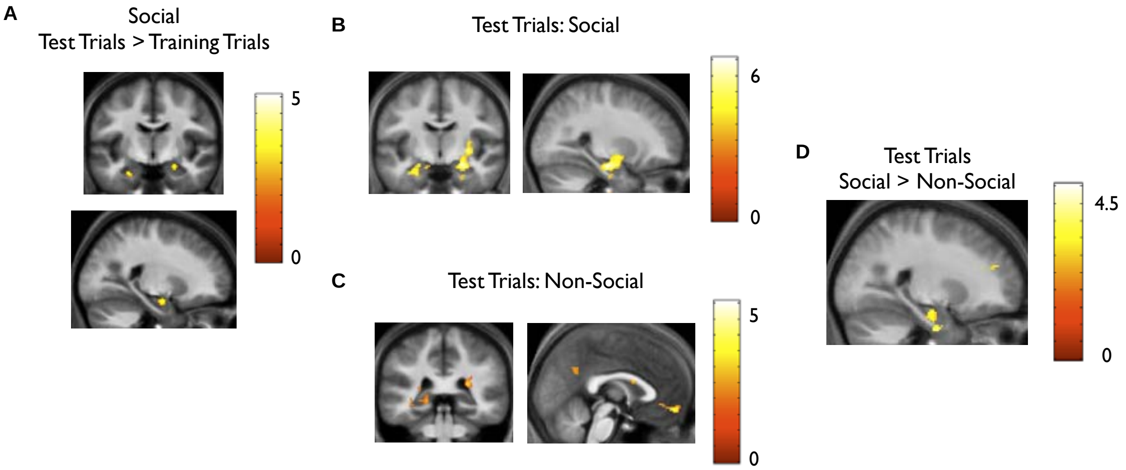

Figure S1: Learn Phase: Result of supplemental analyses where test trial performance was captured solely using the binary performance data (linked to Fig 2)

(A) Activity in the bilateral amygdala/anterior hippocampus, and in no other brain regions, showed a significantly greater correlation with successful performance during test trials, as compared to training trials, in social domain (i.e. at threshold of $p<0.001$ uncorrected and SVC $p<0.05$ corrected: see Supplemental Analysis 2). Note: no above threshold activations were observed in the reverse comparison (i.e. training > test trials).

(B) Activity in the bilateral amygdala/anterior hippocampus showed a robust correlation with the correctness of participants' responses during test trials in social domain (i.e. R amygdala/anterior hippocampus significant at threshold of $p<0.001$ uncorrected and whole brain $p<0.05$ FWE cluster corrected; left amygdala/anterior hippocampus at $p<0.001$ uncorrected and SVC $p<0.05$ corrected - see Supplemental Analysis 3).

(C) Activity in the posterior hippocampus, and vMPFC, correlates with successful performance during test trials in the non-social domain (i.e. at threshold of $p<0.001$ uncorrected and SVC $p<0.05$ corrected).

(D) Activity in the right amygdala, and anterior hippocampus showed a significantly greater correlation with successful performance during test trials in social domain, as

compared to non-social domain (i.e. at threshold of $p<0.001$ uncorrected and SVC $p<0.05$ corrected: see Supplemental Analysis 3).

Activations are displayed on the average structural image of the participants, and thresholded at $p<0.005$ for display purposes.

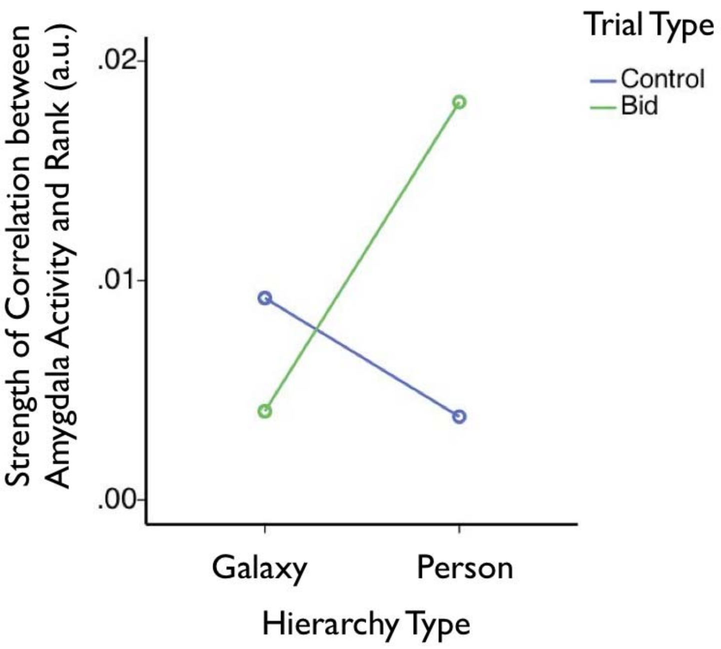

Figure S2. Invest Phase:Activity in Amygdala specifically codes person rank during bid trials: region-of-interest (ROI) analysis. (linked to Fig 7). y-axis: parameter estimates reflecting the strength of the linear correlation between left amygdala activity and rank, with bid trials plotted in green, and control trials in blue. Significant task (bid, control) x hierarchy type (person, galaxy) interaction: see Supplemental Results for details.

Supplementary Table S1. Learn Phase (fMRI): Test Trials (linked to Fig 2).

Table S1A: Social. Brain areas whose activity significantly correlated with the inference score index in the social (i.e. person) condition

Region	*Laterality*	*x*	*y*	*z*	*z-score*
Amygdala	R	20	-4	-18	4.18**
	L	-12	-6	-18	3.88**
Hippocampus (anterior)	L	-20	-18	-22	4.37**
	R	26	-12	-22	4.10**
Hippocampus (posterior) L	L	-30	-36	-2	3.65*
Putamen	R	30	-10	8	3.74**
Insula	L	-56	-8	12	3.89
	R	46	-16	-16	4.27**
Intraparietal sulcus (anterior)	R	38	-16	58	4.42**
Temporoparietal junction	R	50	-64	22	4.03
Lateral orbitofrontal PFC	R	34	38	-6	4.15
Ventromedial PFC	L	-4	52	-12	3.18*
Ventral Striatum	L	-28	8	-8	3.80
Cerebellum	L	-24	-46	-22	3.50
Caudate	L	-10	20	-4	3.35

Table S1B: Social>Non-Social. Brain areas whose activity showed a significantly greater correlation with the inference score index in the social (i.e. person), as compared to the non-social (i.e. galaxy), condition

Region	*Laterality*	*x*	*y*	*z*	*z-score*
Amygdala	R	22	-4	-16	3.13
Amygdala	L	-26	-10	-28	3.45*
	L	-12	-6	-18	3.01*
Hippocampus (anterior)	L	-26	-12	-20	3.32*

All regions are significant at p<0.001 uncorrected for multiple comparisons. All coordinates are in MNI space.

* indicates significant at p<0.05 SVC corrected in regions of a priori interest

** indicates significant at p<0.05 whole brain FWE corrected at cluster level

Supplementary Table S2. Learn Phase (fMRI): Test Trials (linked to Fig 3)

Table S2A: Non-Social. Brain areas whose activity significantly correlated with the correlated with the inference score index in the non-social (i.e. galaxy) condition

Region	*Laterality*	*x*	*y*	*z*	*z-score*
Hippocampus (posterior)	L	-34	-36	-6	3.77*
Ventromedial PFC	R	4	52	-10	3.77*
Ventral Striatum	R	14	10	-10	3.56
Putamen	R	30	-8	4	4.19**
Intraparietal sulcus (anterior)	R	34	-24	50	5.14**
Caudate	R	8	22	-6	4.46**
Insula	R	60	-10	4	4.15

Table S2B. Conjunction: Social & Non-Social. Conjunction (null) analysis showing brain areas whose activity showed a significant correlation with the inference score index in both the social (i.e. person) and the non-social (i.e. galaxy) domains.

Region	*Laterality*	*x*	*y*	*z*	*z-score*
Hippocampus (posterior)	L	-32	-36	-4	3.42*
Intraparietal sulcus (anterior)	R	42	-26	56	4.14**
Ventromedial PFC	R	4	52	-12	3.18*
Insula	R	42	-18	16	3.76
Caudate	R	6	20	-6	3.56
Putamen	R	30	-12	0	3.93

Table S2C: Non-Social>Social. Brain areas whose activity showed a significantly greater correlation with the inference score index in the non-social (i.e. galaxy), as compared to the social (i.e. person), condition

Region	*Laterality*	*x*	*y*	*z*	*z-score*
Insula	L	-34	26	6	3.39

All regions are significant at p<0.001 uncorrected for multiple comparisons. All coordinates are in MNI space.
* indicates significant at p<0.05 SVC corrected in regions of a priori interest
** indicates significant at p<0.05 whole brain FWE corrected at cluster level

Supplementary Table S3. Learn Phase (fMRI): Training Trials (linked to suppl analysis 1).

Table S3A: Social. Brain regions exhibiting a significant correlation between neural activity and successful performance (i.e. probability correct regressor) in social domain.

Region	*Laterality*	*x*	*y*	*z*	*z-score*
Insula	L	-40	0	-12	3.74
Ventromedial PFC	R	8	54	8	3.97
Posterior Cingulate Cortex	R	10	-32	46	3.91

Table S3B. Learn Phase: Functional Neuroimaging Data
Training Trials: Social > Non-Social. Brain regions exhibiting a significantly greater correlation between neural activity and successful performance (i.e. probability correct regressor) in the social, as compared to the non-social, domain.

Region	*Laterality*	*x*	*y*	*z*	*z-score*
Posterior Parietal Cortex	R	48	-66	38	3.45

All regions are significant at $p<0.001$ uncorrected for multiple comparisons. All coordinates are in MNI space.

Supplementary Table 4. Learn Phase: Structural Neuroimaging Data (VBM analyses) (linked to Fig 4)

Table S4A: Social. Brain regions whose gray matter volume shows a significant correlation with participants' capacity to perform transitive judgements of social (i.e. person) rank, indexed by the inference score.

Region	*Laterality*	*x*	*y*	*z*	*z-score*
Amygdala	L	-20	-6	-18	3.18*
	R	20	-3	-18	3.01*
Anterior fusiform gyrus/PHG	L	-30	-8	-33	3.81
Temporal Pole	L	-28	0	-43	3.16
Frontopolar cortex	R	16	61	14	4.25

Table S4B: Social > Non-Social Brain regions whose gray matter volume showed a significantly greater correlation with participants' capacity to perform transitive judgements of social (i.e. person) rank, as compared to non-social (i.e. galaxy) rank.

Region	*Laterality*	*x*	*y*	*z*	*z-score*
Amygdala	L	-20	-6	-18	3.10*
Anterior fusiform gyrus/PHG	L	-30	-8	-33	3.70
Temporal Pole	L	-28	0	-43	3.42
Frontopolar cortex	R	16	61	14	4.19

Table S4C: Non-Social Brain regions whose gray matter volume shows a significant correlation with participants' capacity to perform transitive judgements of non-social (i.e. galaxy) rank, indexed by the inference score.

Region	*Laterality*	*x*	*y*	*z*	*z-score*
Intraparietal sulcus	L	-44	-42	54	3.79
	R	38	-39	50	3.20
Lateral PFC	L	-45	46	-3	3.78
	R	43	54	-1	3.33

All regions are significant at $p<0.001$ uncorrected for multiple comparisons. All coordinates are in MNI space. PHG = parahippocampal gyrus.
* indicates significant at $p<0.05$ SVC corrected in regions of a priori interest

Supplementary Table S5. Invest Phase (fMRI): Bid Trials (linked to Fig 6).

Table S5A: Increasing WTP. Brain areas whose activity showed a significant correlation with participants' WTP (i.e. price they were willing to pay) during bid trials

Region	*Laterality*	*x*	*y*	*z*	*z-score*
Hippocampus (mid)	L	-30	-16	-18	5.05**
Ventromedial PFC	R	8	52	-8	4.08*
	L	-12	54	-8	4.17*
Nucleus Accumbens	R	12	8	-4	4.14**
	L	-10	6	-8	3.46**
Caudate	R	-16	-18	24	4.84**
Posterior Cingulate Cortex	R	12	-40	28	4.08**
Visual Cortex	L	-10	-72	6	4.64**

Table S5B. Main effect of Rank (i.e. collapsed across stimulus type, trial type)**.** Brain areas whose activity showed a significant linear correlation with the rank of the person and galaxy presented during bid trials and control trials.

Region	*Laterality*	*x*	*y*	*z*	*z-score*
Hippocampus (mid)	R	26	-20	-12	4.15 *
	L	-30	-18	-20	3.94*
Hippocampus (posterior)	R	32	-34	-8	4.04*
Amygdala	R	16	-4	-14	3.90**
	L	-30	-8	-18	3.78*
Posterior Cingulate Cortex	R	8	-52	32	5.29**
Ventromedial PFC	R	6	48	-8	3.77**
Medial PFC	R	4	46	18	3.73
Nucleus Accumbens	R	10	10	-12	3.89**
Visual Cortex	L	-16	-94	4	4.86**
Caudate	R	8	16	0	4.93**
Superior temporal sulcus L	L	-48	-36	6	3.93

All regions are significant at p<0.001 uncorrected for multiple comparisons. All coordinates are in MNI space.
* indicates significant at p<0.05 SVC corrected in regions of a priori interest
** indicates significant at p<0.05 whole brain FWE corrected at cluster level

Supplementary Table S6. Invest Phase (fMRI): Bid Trials (linked to Fig 7).

Table S6A: Person Rank. Brain areas whose activity showed a significant linear correlation with the rank of the person presented during bid trials (i.e. increasing activity with higher rank).

Region	*Laterality*	*x*	*y*	*z*	*z-score*
Hippocampus (mid)	R	24	-18	-10	3.41*
Amygdala	R	16	-4	-12	4.34*
	L	-28	-8	-16	3.69*
Ventromedial PFC	R	4	52	-8	3.77**
Posterior Cingulate Cortex	R	4	-50	30	3.90**
Nucleus Accumbens	R	14	10	-6	3.95**
Visual Cortex	L	-16	-70	-10	4.53**

Table S6B: Galaxy Rank. Brain areas whose activity showed a significant linear correlation with the rank of the galaxy presented during bid trials (i.e. increasing activity with higher rank).

Region	*Laterality*	*x*	*y*	*z*	*z-score*
Hippocampus (mid)	L	-30	-24	-18	3.94*
	R	36	-22	-14	3.42*
Ventromedial PFC	L	-10	58	-6	3.46*
Nucleus Accumbens	R	12	8	-6	3.70
	L	-10	16	-8	4.13
Caudate	R	8	4	22	4.29
Posterior Cingulate Cortex	R	12	-42	28	4.10
Insula	R	48	-20	20	4.12
Visual Cortex	L	-18	-88	2	4.75

All regions are significant at $p<0.001$ uncorrected for multiple comparisons. All coordinates are in MNI space.
* indicates significant at $p<0.05$ SVC corrected in regions of a priori interest
** indicates significant at $p<0.05$ whole brain FWE corrected at cluster level

Supplemental Experimental Procedures

Here we provide a full description of experimental procedures (including Appendix detailing task instructions given to participants), and analytic techniques used in the fMRI and voxel-based morphometry analyses.

Participants. Twenty six healthy, right-handed individuals who were currently undertaking or had recently completed a university degree, participated in this experiment (age range 19-31; 12 female). One of these participants failed to fully learn either person or galaxy hierarchies and was therefore excluded from the fMRI analyses. All participants gave informed written consent to participation in accordance with the local research ethics committee.

Stimuli. Pilot experiments informed the selection of face and galaxy stimulus sets that ensured that behavioral performance across social and non-social conditions was equated in both experimental phases. Face pictures were obtained from a widely used database (Stirling database: http://pics.stir.ac.uk): pictures are rendered in grayscale and depict male individuals sitting on a chair, with a neutral expression. Images were cropped below the chin line and resized, though hair was retained to preserve the naturalistic properties of the stimuli. Pictures of galaxies (source: various sites on the internet including http://hubblesite.org/gallery/album/nebula) were chosen to be distinct from one another.

Person and galaxy hierarchies were each comprised of 7 items (i.e. P1-P2-P3-P4-P5-P6-P7 and G1-G2-G3-G4-G5-G6-G7, where P=person and G=galaxy, and 1 is the highest ranking item and 7 the lowest ranking)(Figure 1C). The allocation of individual pictures to position in the hierarchy randomized across the group of participants. In addition, two different face and galaxy pictures were used only during baseline trials. Prior to each scanning session, participants briefly performed a simple 1-back task where they viewed each individual face and galaxy three times – a procedure which is known to minimize stimulus novelty effects during scanning based on previous data (e.g. (Johnson et al., 2008)). Examples of faces and galaxies used are shown in Figure 1.

Tasks and Procedures. Participants were instructed that they would be playing a simple science-fiction computer game, and asked to imagine themselves as an investor in the future (i.e. AD 2100)(see Appendix for full details of task instructions). They were told they would be considering whether to invest in a space mining company which specializes in harvesting a precious mineral (Zircon) from far-away galaxies. They were informed that there would be two parts to the experiment: in the first phase ("Learn" phase) they would need to learn which individuals have more power within the company, and which galaxies have more precious mineral. In phase two ("Invest" phase), they were told that they would need to use knowledge acquired during phase 1 about people and galaxies to decide how much they would be willing to pay (in real monetary terms) for potential projects on offer. Participants were renumerated based on their performance (i.e. % correct responses during the Learn phase) and monetary payout from the Invest phase (see below for details). Our aim, therefore, was to develop a naturalistic experimental scenario where subjects would develop knowledge of a social (i.e. person) and non-social (i.e. galaxy) hierarchy (Learn phase), and subsequently use this information to make prudent investment decisions (i.e. Invest phase).

Phase 1 (Learn) Our experimental task is grounded in the widely acknowledged importance of transitivity to judgements of social rank (Cheney and Seyfarth, 1990; Grosenick et al., 2007; Paz et al., 2004) - and classic implementations of the transitive inference task (Bryant and Trabasso, 1971) (McGonigle and Chalmers, 1977), where dimensions such as length and weight were emphasized (cf mineral content in our study). In this phase of the experiment participants acquired knowledge about the 7-item person and galaxy hierarchies in parallel.

Training trials (Figure 1A). During a training trial, participants viewed adjacent items (people or galaxies) in the hierarchy displayed on either side of the screen (i.e. 6 training pairs: e.g. in person condition: P1 vs P2, P2 vs P3, P3 vs P4, P4 vs P5, P5 vs P6, P6 vs P7). The left-right position of an item on the screen was randomized across trials. They had 3 seconds in which to choose, via button press (i.e. left or right, index or middle finger of right hand respectively), the item which had "more power" (person condition) or

"more mineral" (galaxy condition). After 3 seconds, a feedback screen appeared: this consisted of a green square border which indicated the participant's choice together with either "+20 points" or "-20 points", for a correct or incorrect response respectively. A fixation cross of 1.5 seconds duration preceded the onset of the next trial. The renumeration received by participants for this phase of the experiment was determined directly from the number of points won.

Test trials (Figure 1B). During test trials, participants viewed pairs of non-adjacent items in the hierarchy (i.e. 6 inference pairs, e.g. in person condition: P2 vs P4, P2 vs P5, P2 vs P6, P3 vs P5, P3 vs P6). As in training trials, participants had 3 seconds in which to choose, via button press (i.e. left or right), the item which they thought had more power (person condition) or more mineral (galaxy condition). Importantly, however, no feedback was presented during test trials, though participants were instructed that their choices would still count towards their final payout. Instead, after 3 seconds, a screen appeared which required participants to rate (on a scale of 1 to 3) their confidence in their decision: participants were carefully instructed to enter a "1" response if they were guessing entirely, a "2" response if they were "had some idea but were not sure" about their choice, and to reserve a "3" response until they were "more than 90% certain" that their choice was the correct one. Participants were told that though their confidence responses would not count towards their final payout, they should still answer as accurately as possible.

Inference score index: as outlined previously, this trial-by-trial measure of transitivity performance, which was validated in a separate behavioral experiment (see Supplemental Results), was designed to provide leverage on the level of hierarchical knowledge attained at a given timepoint during the Learn phase. It was derived by combining (i.e. multiplying) the correctness of participants response with their confidence rating (range 0-3). In constructing this index, we assumed that for correct responses, the strength of memory representations of the hierarchy should vary as a function of participants' confidence in their choice. Whilst incorrect responses were assumed to reflect a weaker memory strength (cf correct responses), we had no strong prediction that this would vary

as a function of confidence. Based on these intuitions, the inference score was constructed by multiplying the correctness of the binary choice, scored as 1 or 0, by the confidence rating – resulting in a scale that ranged from 0 to 3. Importantly, we validated the inference score index in an independent behavioral study prior to the fMRI experiment (see Supplemental Results), which provides objective evidence of its explanatory value by linking it to participants' ability to accurately state the rank order of items, a direct test of relational knowledge of the hierarchy (e.g. Smith and Squire, 2005.

It is worth noting that one could make a different starting assumption about the relationship between the measured variables (i.e. correctness, confidence) and underlying memory strength – specifically, that higher confidence ratings reflect stronger memories for *both* correct and incorrect responses, resulting in a scale that varies from -3 to +3 (i.e. formed through the multiplication of the correctness of the binary choice by the confidence rating – but with incorrect responses scored as -1 rather than zero). In fact, these two alternative schemes for constructing the inference score index would yield highly correlated measures in our experiment. Further, an additional analysis using this alternative inference score index (i.e. ranging from -3 to +3) in the fMRI analyses resulted in a highly similar pattern of findings for the reported contrasts.

Baseline trials: These trials were designed to be similar to training trials in terms of visual display, requirement for motor response, reward (i.e. presence of positive and negative monetary feedback), but without requiring participants to learn associative information. The timeline of baseline trials was analogous to learning trials. However, an asterisk always appeared below one of the faces or galaxies indicating to the participants which button they should depress.

Schedule of trial presentation.

Blocks of Person trials alternated with blocks of Galaxy trials, with block order (i.e. whether person or galaxy condition appeared as the first block) counterbalanced across subjects. Each block was comprised of a 20 trial miniblock made up of 12 training trials

with 2 baseline (i.e. baseline) trials interspersed, followed by a 6 trial miniblock of test trials. The order of training and test trials was pseudorandomized and varied across blocks. The start of each miniblock was preceded with the relevant instruction which was presented for 7 seconds (i.e. “Get ready for Person Training trials”, “Get ready for Person Test trials”). In total, there were 15 blocks for each of the Person and Galaxy conditions – i.e. 180 training trials, 30 baseline trials, and 90 test trials, in each condition. Phase one consisted of three sessions of approximately 20 minutes each, separated by a 1 minute break during which time participants remained inside the MRI scanner.

Phase 2 (Invest). In this phase of the experiment, participants were required to use their knowledge about person and galaxy hierarchies to decide a) how much in real monetary terms to pay for potential projects on offer (“bid” trials: Figure 5A), by evaluating the potential worth of individual people and galaxies based on their rank or b) which item (i.e. person or galaxy) was more highly ranked, and by how much (“control” trials: Figure 5B).

Bid trials. Participants were instructed that in this type of trial they would be required to declare the maximum amount of money they would be willing to pay (i.e. WTP) to purchase shares in potential projects on offer. A project was said to consist of the combination of a particular person and a particular galaxy, and participants told to imagine that this person would be heading up a mission to go to this galaxy to harvest mineral.

On a given trial, the screen displayed one person and one galaxy (e.g. P4 and G2: Figure 2), with the left-right location randomized between trials. All 49 person-galaxy combinations were presented in different trials, with two repetitions of each. As such, person rank and galaxy rank were orthogonalized by experimental design, allowing us to isolate brain regions showing a (linear) correlation with rank for each stimulus type. Participants had 8 seconds in which to declare their WTP by moving a cursor (leftward motion: index finger, rightward: middle finger) to the desired position on a continuous scale from zero to twenty pounds. Participants confirmed their bid (i.e. WTP) using their ring finger, which caused the cursor to change color from white to red.

Participants received detailed instructions on all aspects of the bid task (see Appendix for details). They were told that the actual worth of the shares was determined directly by the rank of the person and galaxy involved, and that the rank of each stimulus type was equally important. Participants were given examples of the actual worth of person/galaxy combinations (e.g. the highest ranking person together with the highest ranking galaxy would have an actual worth of essentially £20); they were *not* however explicitly informed that the relationship between person/galaxy rank and actual worth was linear, and determined by the following function:

actual worth (£) = $20 – ((R_p - 1)+ (R_g -1))*k$

Where R_p and R_g denote the rank of person and galaxy, respectively, from 1 (high) to 7 (low), and k denotes the price increment for each unit change in rank, and is equal to 1.67 (i.e. £20 / (2*6)). For instance, the actual worth of a project involving P1 and G2 would be: 20 – 1.67=£18.23.

2) Participants were told that they would be playing with £20 from their winnings from phase 1 of the experiment. Whilst they would place bids on many trials, only one trial (e.g. that involving P2 and G3) would be randomly selected to be played out as a real money transaction at the end of this phase of the experiment. As such, they would not need to spread the £20 over multiple trials, but could treat each trial as if it was the only one.

3) The real money transaction was played out as a Becker-DeGroot-Marshak (BDM) auction, a widely used incentive-compatible mechanism in behavioral economics(Becker et al., 1964), and neuroeconomics (e.g.(Plassmann et al., 2007
)) for ensuring that participants' prices reliably reflect what they actually think a given option is worth. Participants received detailed instruction as to the workings of the BDM mechanism. Care was taken to ensure that participants understood that the optimal strategy in bid trials was to state a WTP that was close as possible to the actual worth of

shares in the project - and that assuming they adopted the optimal strategy, then the expected payout (i.e. expected value, EV) of a given trial would be greater for higher ranking people and galaxies (i.e. highest for a trial involving P1 and G1).

Several illustrative scenarios were described (see Appendix for details): participants were told that for the selected trial (e.g. involving person 2 and galaxy 3), the market would issue shares at a random price (between £0 and 20, uniform distribution). If this randomly generated BDM price (termed “market issue” price e.g. £7) was lower than the maximum they were willing to pay for the shares (i.e. WTP: e.g. £12), then they would purchase the shares at the market price (i.e. £7). Having purchased the shares, they would then sell the shares at their actual worth, in this case amounting to £15.00 (i.e. determined by the linear function relating rank to actual worth described above). In this case, they were told they would receive a net profit of: actual worth – BDM price = £8.00.

On the other hand, participants were told that if the market price was higher than their WTP, then the transaction would not proceeed (i.e. they would not purchase the shares), and therefore they would neither win or lose money from this section of the experiment.

Control trials. Control trials were designed to closely match bid trials in terms of task demands (i.e. retrieval of hierarchical knowledge, motor requirements, and overall performance (see Supplemental Results: regression analyses). In control trials, however, participants were not required to evaluate the value of an investment project based on the worth of individual people and galaxies (i.e. as in bid trials)- but rather determine which of the two items were relatively higher in rank, and by how much, in a more abstract context. During control trials, therefore, the expected payout from a given trial was effectively unrelated (i.e. orthogonal) to the rank of the person and galaxy presented (with participants rewarded according to accuracy, up to a maximum of £20). In contrast, highly ranking items were of greater motivational significance during bid trials, with the expected payout of a given trial directly dependent upon the rank of the items (i.e. person, galaxy) presented.

As in bid trials, control trials involved the presentation of a particular person-galaxy combination (e.g. P6 G4), with left-right locations randomized across trials. All 49 person-galaxy combinations were presented in different trials, with two repetitions of each: as before, person rank and galaxy rank were orthogonalized by experimental design, allowing us to isolate brain regions showing a (linear) correlation with rank for each stimulus type. Participants were required to position the cursor (using index, middle and ring fingers for leftward, rightward, and confirm actions respectively) on a continuous scale according to which item (i.e. person or galaxy) was higher in its respective "pecking order", and by how much. Rather than indicating monetary amounts (i.e. as in Bid trials), the scale ranged from "galaxy higher" on its leftmost aspect to "person higher" on its rightmost aspect. As for the Bid trials, participants were given illustrative examples to ensure they understood their task in these trials, and informed that at the end of the experiment one trial would be randomly selected to count towards their monetary payoff (up to a maximum of £20): whilst no monetary transaction was to take place, participants were aware that they would be paid according to their accuracy in the selected control trial (i.e. calculated as the difference between their chosen cursor position and objectively correct cursor position).

Bid trials and control trials were presented within blocks (7 trials each), with presentation order randomized across participants. 98 trials of each type were divided over 2 experimental sessions lasting approximately 20 minutes each. Participants had a 1 minute break between sessions during which time they remained inside the scanner.

Post-Experimental Debriefing (after completion of Phase 2). Participants were carefully debriefing following the end of phase 2 of the experiment. Included in this assessment was a test assessing participants' declarative knowledge of the hierarchy: pictures of the set of people and galaxies were presented to participants, and they were asked to rank them in terms of their order in the hierarchy, with their performance timed.

Social realism score: Participants were also asked to evaluate how "real" the social rank dimension seemed:

"In phase 2, when you saw a picture of an individual who was more highly ranked in the company, how "real" did it seem that they were more highly ranked or had more power in the company etc? Please rate this on a scale of 1-10 (10 = a lot, 1 = not at all)- as an example, if when you saw the most highly ranked guy you thought to yourself that's the topdog/head-guy, then your answer is likely to be nearer the 10 end of the scale"

Face ratings: participants were asked to rate the trustworthiness and attractiveness of the face stimuli: i.e. "How x is this person? (1=not at all, 9=extremely). Use your first impressions."

Behavioral analyses. Analyses were conducted using SPSS software (www.spss.com), Matlab 7.0 (www.mathworks.com/products/matlab), and using the state-space model (see below) toolbox obtained from www.neurostat.mit.edu.

fMRI design. The temporal pattern of stimulus presentation was designed to maximise statistical efficiency whilst preserving psychological validity, in line with established procedure (Frackowiak et al., 2004; Friston et al., 1998; Josephs and Henson, 1999). Importantly, the haemodynamic response to events that occur a few seconds apart is explicitly modelled (via a haemodynamic response function), and therefore can be estimated separately for each event type by implementing the general linear model as is standard when using statistical parametric mapping software (SPM8) (www.fil.ion.ucl.ac.uk/SPM) (also see below) (Friston et al., 1998).

Functional imaging acquisition parameters. T2 weighted gradient-echo planar images (EPI) with BOLD (blood oxygen level dependent) contrast were acquired on a 3.0 tesla Siemens Allegra MRI scanner using a specialized sequence to acquire whole brain coverage, whilst minimizing signal dropout in the medial temporal lobe and ventromedial prefrontal cortex(Weiskopf et al., 2006). We used the following scanning parameters to achieve whole brain coverage: 48 oblique axial slices angled at 30^0 in the anterior-posterior axis, TR 2.88 seconds, TE 30ms, 2mm thickness (1mm gap), in-plane resolution 3x3 mm, z-shim -0.4mT/m*ms, negative phase encoding direction. During phase 1 of the

experiment, 3 runs of 479 volumes were acquired. 2 runs of 393 volumes were acquired during phase 2. High-resolution (1x1x1mm) T1-weighted structural MRI scan were also acquired for each participant after functional scanning. These were coregistered to the functional EPIs, and averaged across participants to aid localization.

fMRI data preprocessing. Images were analyzed in a standard manner using the statistical parametric mapping software SPM8 (www.fil.ion.ucl.ac.uk/SPM). After the first six “dummy volumes” were discarded to permit T1 relaxation, EPI images were spatially realigned and unwarped using fieldmaps(Andersson et al., 2001), followed by spatial normalization to a standard EPI template. Normalized images were smoothed using a gaussian kernel with full width at half maximum of 8mm.

Phase 1 (Learn) fMRI data analysis. Following preprocessing, the event-related fMRI data were analyzed in SPM8 using the general linear model (GLM) following established procedures (Frackowiak et al., 2004; Friston et al., 1998). We targeted our analyses to detect brain regions whose activation pattern during test trials significantly correlated with participant-specific trial-by-trial parametric regressors.

We focused our fMRI analyses on test trials, because it was here that successful performance in our paradigm was driven primarily by knowledge of the hierarchy, with the inference score index providing an online index charting the level of such knowledge across the experimental phase (see above). In contrast successful training trial performance in our paradigm - as well as transitive inference studies more generally (e.g. reviewed in (Zeithamova et al., 2012)) – can be achieved simply by memorizing the correct item in each pair, and therefore does not require knowledge about the hierarchy. Indeed, rodents with hippocampal damage show performance on trained trials that is indistinguishable from control animals, despite markedly impaired transitivity performance (e.g. Dusek and Eichenbaum, 1997).

Specification of first-level design matrix.

Test trials. As a first step, the 5 second period during which item pair and confidence rating were displayed during test trials was modeled as a boxcar function and convolved with the canonical haemodynamic response function (HRF) to create regressors of interest. All test trial types (i.e. 6 pairs: P2 vs P4, P2 vs P5, P2 vs P6, P3 vs P5, P3 vs P6, P4 vs P6) were modeled within these regressors, with one regressor for the person condition and one for the galaxy condition.

The following participant-specific vectors were then included as parametric modulators in the design matrix (in order): 1) **trial-by-trial reaction time** (RT) 2) **probability correct**: trial-by-trial estimates of the probability of a correct response derived from learning curves, constructed separately for each of the 6 test pairs (e.g. P2 P6) by the state-space model (see(Smith et al., 2004) for a detailed description). The state-space model(Smith et al., 2004) www.neurostat.mit.edu, is a technique which computes an estimate of the learning curve (i.e. probability of correct response as a function of trial number) from the sequence of binary correct/incorrect responses, using an expectation maximisation (EM) algorithm(Smith et al., 2004). This technique has previously used to correlate neural activity with binary performance data during learning experiments in monkeys (Wirth et al., 2003) and fMRI (Kumaran et al., 2009; Law et al., 2005). 3) **inference score index** : a trial-by-trial measure (see above): where 3 points indexed correct responses given a "very sure" confidence rating, 2 points a correct response given a "some idea" rating, and 1 point for a correct responses afforded a "guess" rating. Incorrect responses were scored as 0.

These parametric regressors were also convolved with the HRF, leading to the height of the HRF for a given event being modulated accordingly. Thus, these regressors model BOLD signal changes that covary with specific behavioral indices of performance on a given trial (e.g. inference score during test trials). Note that, the automatic serial orthogonalization procedure carried out by SPM8 results in shared variance among regressors being captured by earlier regressors. This procedure, therefore, allows one to ask in which brain regions neural activity specifically tracks the emergence of

hierarchical knowledge, indexed by the inference score regressor, and cannot be explained by non-specific changes in RT with learning or the effects of an overall improvement in performance.

Training trials. As a first step, the 5 second period during which item pair and outcome was displayed during training trials was modeled as a boxcar function and convolved with the canonical haemodynamic response function (HRF) to create regressors of interest. All training trial types (i.e. 6 pairs: P1 vs P2, P2 vs P3....P6 vs P7) were modeled within these regressors, with one regressor for the person condition and one for the galaxy condition.

The following participant-specific vectors were then included as parametric modulators in the design matrix (in order): 1) **trial-by-trial reaction time (RT)** 2) a **binary performance vector** consisting of 1s for correct and 0s for incorrect responses respectively 3) **probability_correct** vector: trial-by-trial estimates of the probability of a correct response derived from learning curves, constructed separately for each training trial type (e.g. P1 vs P2), using the state space model (see above).

As outlined previously, these parametric regressors were also convolved with the HRF, leading to the height of the HRF for a given event being modulated accordingly. This procedure, therefore, allows one to ask in which brain regions neural activity specifically tracks proficient performance during training trials, indexed by the probability correct regressor, and cannot be explained by non-specific changes in RT with learning or the effects of reinforcing feedback.

We also included vectors coding for baseline trials, in the first level design matrix. Further, participant-specific movement parameters were included as regressors of no interest. A high pass filter with a cutoff of 180 seconds was employed. Temporal autocorrelation was modelled using an AR(1) process.

Phase 2 (Invest). fMRI data analysis. Following preprocessing, the event-related fMRI data were analyzed in SPM8 using the general linear model (GLM) following established procedures (Frackowiak et al., 2004; Friston et al., 1998). We set up two different parametric models to detect brain regions whose activation pattern 1) exhibited a significant linear correlation with the maximum amount of money participants were willing to pay for shares in a project during bid trials (i.e. WTP) 2) showed a significant linear correlation with the rank of person or galaxy in the hierarchy, during bid or control trials. Two statistical models were used since by experimental design the WTP for a given trial was a direct function of the rank of items displayed: as such including a WTP parametric regressor in the same model as the person (or galaxy) rank parametric regressor would lead to a substantial reduction in statistical power for detecting the relevant effects (i.e. relating to person and galaxy rank coding).

Specification of first-level design matrix. In both models, the 8 second trial period during which the person/galaxy combination was displayed on the screen during bid and control trials, and participants made their response, was modeled as a boxcar function and convolved with the canonical haemodynamic response function (HRF) to create regressors of interest.

fMRI parametric model one. The following vectors were then included as parametric modulators in the design matrix (in order): 1) **trial-by-trial reaction time** (RT) 2) **WTP**: participants' stated maximum price that they were willing to pay for the shares in the project.

fMRI parametric model two. The following vectors were then included as parametric modulators in the design matrix (in order): 1) **trial-by-trial reaction time** (RT) 2) **galaxy rank**: from 1 to 7, linear and quadratic components modeled. 3) **person rank**, from 1 to 7, linear and quadratic components modeled. As mentioned previously, galaxy rank and person rank are orthogonal by experimental design (i.e. all 49 combinations of person and galaxy were presented during Bid and Higher trials), which allowed us to identify brain

regions showing a specific correlation between neural activity and Rank for each stimulus type.

In a supplemental analysis, post-scan ratings of attractiveness and trustworthiness were also entered as additional parametric regressors in the first level model, prior to the person rank parametric regressor.

These parametric regressors were also convolved with the HRF, leading to the height of the HRF for a given event being modulated accordingly. Thus, these regressors model BOLD signal changes that covary with specific indices on a given trial (e.g. the rank of a person). Further, participant-specific movement parameters were included as regressors of no interest. A high pass filter with a cutoff of 180 seconds was employed. Temporal autocorrelation was modelled using an AR(1) process.

"Illustrative" Model: The parametric models specified above were used for statistical inference- i.e. to ask which brain regions show a significant linear correlation between the amplitude of neural activity and person/galaxy rank. In contrast, this illustrative model, which included separate regressors for each person and galaxy rank in both bid and control conditions (i.e. 28 regressors of interest in total), was used solely to graphically represent the linear relationship between neural activity in a given brain region (e.g. amygdala) and person rank (see Figure 7; also see(Winston et al., 2002) for a similar useage).

Model Estimation. Model estimation proceeded in two stages. In the first stage, condition-specific experimental effects (parameter estimates, or regression coefficients, pertaining to the height of the canonical HRF) were obtained via the GLM in a voxel-wise manner for each participant. In the second (random-effects) stage, participant-specific linear contrasts of these parameter estimates, collapsed across the three sessions, were entered into a series of one-sample t tests [as is standard when using SPM(Frackowiak et al., 2004)], each constituting a group-level statistical parametric map.

Statistical inference.

Voxel-based analyses. ***Voxel-based analyses***. We report results in a priori regions of interest - the hippocampus, amygdala and ventromedial prefrontal cortex - where activations are significant at $p<0.001$ uncorrected for multiple comparisons, and survive small volume correction (SVC) for multiple comparisons (at $p<0.05$ corrected) using SPM8. For the SVC procedure we used anatomical masks, for the hippocampus and amygdala, traced over the average structural image of the participants. The amygdala was outlined based on established criteria described in: ((Brierley et al., 2002). For the vMPFC we used an 8mm sphere centred on coordinates derived from a previous related study(Kumaran et al., 2009):[x, y, z = -4 52 -14].

Activations in other brain regions were only considered significant if they were significant at a level of $p<0.001$ uncorrected, and additionally survived whole brain FWE correction at the cluster level ($p<0.05$ corrected), in line with established procedures(Frackowiak et al., 2004) - but are reported for completeness at a threshold of $P<0.001$ uncorrected for multiple comparisons.

All activations are displayed on sections of the average structural image of all the participants. Reported voxels conform to MNI (Montreal Neurological Institute) coordinate space. Right side of the brain is displayed on the right side.

Conjunction analysis. We performed a conjunction "null" analysis as implemented in SPM8, which ensures that each individual contrast (i.e. brain regions whose activity correlates with the inference score index during test trials in the social condition *and* the non-social condition) was individually significant (at a threshold at $p<0.001$ uncorrected for multiple comparisons)(Friston et al., 2005). As for the main fMRI analyses, activations were considered significant in regions of interest (see above) if they survived a SVC correction at $p<0.05$.

Region of Interest (ROI) analyses. We performed a functionally defined ROI analysis (using the MarsBar SPM toolbox: http://marsbar.sourceforge.net/) to ask whether the

amygdala showed a significantly greater linear correlation with person rank, as compared to galaxy rank, during bid trials. Regions in the left amygdala, as well as comparison regions (the left hippocampus and vMPFC) were functionally defined from the group statistical map pertaining to brain regions showing a linear correlation with rank, collapsed across stimulus type (person and galaxy) and task (bid and control), thresholded at $p<0.001$ uncorrected (i.e. the main effect of rank: Table S5B). Using the MarsBar SPM toolbox, we obtained parameter estimates for all voxels within this region, for the group as a whole. These parameter estimates were averaged across the ROI and entered into a repeated measures ANOVA with factors: brain region, task (bid, control) and hierarchy type (person, galaxy). It is important to note that these analyses treat data from a ROI as if it was from a single voxel and hence no correction for multiple comparisons is necessary. Results, therefore, were considered statistically significant where they pass a threshold of $p<0.05$.

Selection contrast is unbiased with respect to contrasts of interest. ROI analyses are widely held to be a powerful tool for affording additional insights, above and beyond that provided by univariate fMRI analysis(Kriegeskorte et al., 2009). Recent work has highlighted potential shortcomings of previous work, and established a theoretically principled approach for carrying out an ROI analysis. Importantly, our analysis fulfils the criteria outlined by Kriegeskorte et al (2009): the definition of these ROI is *unbiased*, and therefore statistically independent, with respect to the contrasts relevant to addressing our two experimental questions of interest concerning the amygdala(Kriegeskorte et al., 2009). Specifically, the main effect contrast used to define the ROIs is orthogonal to the relevant contrasts of interest (i.e. their matrix dot product equals zero). Further, all other parameters were balanced across the two tasks and stimulus types (i.e. person, galaxy), for example the number of experimental trials, preventing other sources of bias entering into the analysis.

Voxel-Based Morphometry (VBM) Analysis.

Structural MRI data acquisition and preprocessing. VBM is an analytic technique which allows regional volumetric differences in brain structure between participants to be

characterized(Ashburner, 2007; Ashburner and Friston, 2000; Kanai and Rees, 2011). A 3.0T Allegra scanner was used to acquire high resolution T1-weighted whole brain scans (parameters to be added: 176 slices, echo time=3.56ms, TR=12.24ms, voxel size=1mm isotropic). To maximize the size of the group, all 26 participants who took part in the experiment were included in the VBM analysis. Note however, that all effects reported remained robust (as detailed in the Supplemental Results) when only the 25 participants included in the main fMRI analyses were included.

Image preprocessing was conducted using SPM8 and allows gross morphological differences between participants to be removed whilst preserving regional gray matter volumes. As a first step, structural T1-weighted scans were segmented into cerebrospinal fluid, white matter, and gray matter (GM) in native space. Then, optimized intersubject image registration was performed using the DARTEL (diffeomorphic anatomical registration through exponentiated lie algebra) toolbox(Ashburner, 2007). DARTEL effects an iterative process by individual GM segment images are rigidly aligned and matched to an improving average template image across participants. Next, smoothed normalized images were generated using DARTEL's "normalize to MNI space" module: individual GM images were transformed using a transformation specified by the affine registration of the DARTEL template generated in the previous step to MNI space, and DARTEL flow fields. This procedure ensures that local tissue volumes are preserved after the registration setup (i.e. equivalent to a Jacobian modulation step). Smoothing was performed with a Gaussian kernel of 8mm full width to half maximum. Preprocessed GM images were entered into a general linear model using SPM8, with adjustments made for total intracranial volume using proportional scaling. A binary mask (SPM8 grey.nii template > 0.3) was used to restrict the search volume to GM changes.

We performed a whole-brain voxel-wise analysis to examine the relationship between gray matter volume across the brain and behavioral performance on the hierarchy learning task (i.e. phase 1). T statistic maps were generated to reflect the correlation between behavioral measures (e.g. inference score) and regional GM volume, with effects reported as significant based on criteria defined previously for the fMRI analyses: i.e.

effects are considered significant in regions of interest at $p<0.001$ uncorrected for multiple comparisons, where they survive small volume correction (SVC) for multiple comparisons using anatomical masks (see above). For other regions, a significance threshold of $p<0.05$ family wise error (FWE) correction across the whole brain was applied.

Region of Interest (ROI) analysis. Mean gray matter volume was calculated for the amygdala as a whole, defined by an anatomical mask traced over the average structural image of the participants based on previously outlined criteria(Brierley et al., 2002), using the MarsBar SPM toolbox (http://marsbar.sourceforge.net/). As mentioned previously, ROI analyses treat data from a single region of interest (e.g. amygdala) as if it was from a single voxel and hence no correction for multiple comparisons is necessary. Correlations observed in these analyses, therefore, are reported as statistically significant where they pass a threshold of $p<0.05$.

Supplemental References.

Andersson, J.L., Hutton, C., Ashburner, J., Turner, R., and Friston, K. (2001). Modeling geometric deformations in EPI time series. Neuroimage 13, 903-919.

Ashburner, J. (2007). A fast diffeomorphic image registration algorithm. Neuroimage 38, 95-113.

Ashburner, J., and Friston, K.J. (2000). Voxel-based morphometry--the methods. Neuroimage 11, 805-821.

Becker, G., DeGroot, M., and Marshak, J. (1964). Measuring Utility by a Single Response Sequential Method. Behav Sci 9, 226-232.

Brierley, B., Shaw, P., and David, A.S. (2002). The human amygdala: a systematic review and meta-analysis of volumetric magnetic resonance imaging. Brain Res Brain Res Rev 39, 84-105.

Fleming, S.M., Dolan, R.J., and Frith, C.D. (2012). Metacognition: computation, biology and function. Philos Trans R Soc Lond B Biol Sci 367, 1280-1286.

Fortin, N.J., Wright, S.P., and Eichenbaum, H. (2004). Recollection-like memory retrieval in rats is dependent on the hippocampus. Nature 431, 188-191.

Frackowiak, R.S., Friston, K.J., Frith, C.D., Dolan, R.J., Price, C.J., Zeki, S., Ashburner, J., and Penny, W. (2004). Human Brain Function.

Friston, K.J., Fletcher, P., Josephs, O., Holmes, A., Rugg, M.D., and Turner, R. (1998). Event-related fMRI: characterizing differential responses. Neuroimage 7, 30-40.

Friston, K.J., Penny, W.D., and Glaser, D.E. (2005). Conjunction revisited. Neuroimage 25, 661-667.

Guderian, S., Brigham, D., and Mishkin, M. (2011). Two processes support visual recognition memory in rhesus monkeys. Proc Natl Acad Sci U S A 108, 19425-19430.

Johnson, J.D., Muftuler, L.T., and Rugg, M.D. (2008). Multiple repetitions reveal functionally and anatomically distinct patterns of hippocampal activity during continuous recognition memory. Hippocampus.

Josephs, O., and Henson, R.N. (1999). Event-related functional magnetic resonance imaging: modelling, inference and optimization. Philos Trans R Soc Lond B Biol Sci 354, 1215-1228.

Kanai, R., and Rees, G. (2011). The structural basis of inter-individual differences in human behaviour and cognition. Nat Rev Neurosci 12, 231-242.

Kriegeskorte, N., Simmons, W.K., Bellgowan, P.S., and Baker, C.I. (2009). Circular analysis in systems neuroscience: the dangers of double dipping. Nat Neurosci 12, 535-540.

Law, J.R., Flanery, M.A., Wirth, S., Yanike, M., Smith, A.C., Frank, L.M., Suzuki, W.A., Brown, E.N., and Stark, C.E. (2005). Functional magnetic resonance imaging activity during the gradual acquisition and expression of paired-associate memory. J Neurosci 25, 5720-5729.

McGonigle, B.O., and Chalmers, M. (1977). Are monkeys logical? Nature 267, 694-696.

Plassmann, H., O'Doherty, J.P., and Rangel, A. (2007). Appetitive and aversive goal values are encoded in the medial orbitofrontal cortex at the time of decision making. J Neurosci 30, 10799-10808.

Smith, A.C., Frank, L.M., Wirth, S., Yanike, M., Hu, D., Kubota, Y., Graybiel, A.M., Suzuki, W.A., and Brown, E.N. (2004). Dynamic analysis of learning in behavioral experiments. J Neurosci 24, 447-461.

Squire, L.R., Wixted, J.T., and Clark, R.E. (2007). Recognition memory and the medial temporal lobe: a new perspective. Nat Rev Neurosci 8, 872-883.

Weiskopf, N., Hutton, C., Josephs, O., and Deichmann, R. (2006). Optimal EPI parameters for reduction of susceptibility-induced BOLD sensitivity losses: a whole-brain analysis at 3 T and 1.5 T. Neuroimage 33, 493-504.

Wirth, S., Yanike, M., Frank, L.M., Smith, A.C., Brown, E.N., and Suzuki, W.A. (2003). Single neurons in the monkey hippocampus and learning of new associations. Science 300, 1578-1581.

Zeithamova, D., Schlichting, M.L., and Preston, A.R. (2012). The hippocampus and inferential reasoning: Building memories to navigate future decisions. Front Human Neurosci.

APPENDIX: Instructions for the Space Mining Task

You are asked to imagine you are investor in the future (AD 2100). You are considering investing in a space mining company whci specializes in harvesting precious minerals from far away galaxies. There will be 2 parts to the experiment: in the first part, you will need to learn two things (through trial and error)- 1) which galaxies have more precious **mineral** (called Zircon) 2) which individuals have more **power** in the company. In the second phase of the experiment, you'll need to use this knowledge about galaxies and individuals to decide whether to invest in projects on offer.

LEARN PHASE (I). On **training** trials, you will see pairs of galaxies, or people, and need to learn which has more precious mineral, or has more power in the company. Your job is to collect points by choosing the correct one (pressing right or left keys). You'll win 20 points for choosing the right one, and lose 20 for choosing the wrong one. You will be given a limited time in the actual experiment and see different pictures. In the next screen, a box will indicate your choice- and below this will be an indication of whether you were correct or incorrect. Of course you'll not know which is the correct one to start with i.e. you have to guess – but you should be able to learn through feedback (though not an easy task, so do your best). And even at the start, better to guess than not to answer (since 50% if guess, but definitely wrong if don't answer). Important to do your best throughout since you will be paid according to performance. Don't worry it's a difficult task- so just do your best! Note you will also see "easy trials" (baseline) and all you need to do is just press the button corresponding to the side of the star. Now I want to tell you about the **test trials:** these are similar to training trials- i.e. you will need to choose which galaxy has more Zircon, or which person has more power, but no feedback is provided. Note, however, that your responses count just as training trials for computing your pay. You'll notice that there will be pairs presented together that aren't presented during training trials- here you'll have to use your judgement to choose the correct one (you might find these trials difficult particularly early on). You will also be asked to rate your confidence in your choices during test trials on a scale of 1-3: we'd like you to answer 1 if you are literally guessing, 2 if you have some idea that your choice is the right one- only answer 3 if you are really very sure that your choice is correct (i.e. more than "90% sure" that your choice is the correct one. Though your confidence responses don't count towards the pay, it's important for our analysis that you answer truthfully.

INVEST PHASE (II) There are 2 types of trials: in **Bid trials** you will be playing with £20 of your winnings from the previous session and have the opportunity to win more (up to a maximum of a further 20)- but you'll have to play well! In bid trials you will be presented with potential projects (consisting of a person/galaxy pair), and decide the maximum amount you are willing to pay for shares relating to this project. You can imagine that it's as if this person is heading up a mission to go and harvest mineral from this galaxy. To give you a real world example- consider this pen-you might say that the maximum you're willing to pay for this is £1. This means that you'd be happy to get it for £0.90, or even £0.99, but you wouldn't buy it at £1.01. You'll be doing exactly the same kind of thing in this task. So on each trial, you'll need to place the cursor (note the cursor appears at random starting position which doesn't convey any useful information) at the position according to the maximum you would pay for shares in this project – scale is from 0 to £20 in real money terms to you- in this fictitious world, you could think of it as corresponding to thousands of pounds. You move the cursor by holding down left and right buttons, and you'll also need to confirm your price using this third button- important to do this, and after you've confirmed you can't change your mind (you'll have 8 secs- so just do it as fast as you're able and remember to confirm the price).

Now I want to tell you a few things: firstly-the actual worth of shares is determined directly by the rank of person and galaxy- for example, a project involving the highest ranked person and

galaxy would be worth effectively £20, and the combination of the lowest ranked person and galaxy would be worth basically zero- other projects will be somewhere in between these extremes- e.g. a middle ranking person and galaxy containing an average amount of mineral would be something like £10. Note also that the rank of galaxy and person are equally important in determining share worth. Of course, I'm sure you appreciate you'll need to use previous knowledge to determine how much shares in these projects are actually worth.What's important is that you actually put the cursor at the maximum amount you are willing to pay for shares, because at the end of this phase, we'll actually play out 1 of these transactions (i.e. trials) for real money. Which transaction it'll be is randomly determined at the end of the experiment- I don't know which one it'll be and nor do you (it's randomly determined by a computer program)- so it's important to treat every single trial as if it were the one that will count

Now what I want to do is to take one trial as an example: let's imagine you said you would pay up to a max of £15 for the shares- and when you come out of the scanner, this trial gets selected to play out as a real money transaction. What happens is that the market issues these shares at a price- and this price is actually random and can fall anywhere between 0 and £20 with equal probability. If the market issue price is above your maximum price, then no transaction occurs-as such, you don't win or lose money from this bit of this phase of the experiment. But if the market price is below your max price – i.e. it is below £15 -say £5- you'll actually buy these shares from us at the market price- indeed you should be happy to buy at £5 since you said you'd be happy to pay up to a max of £15- so what happens is you effectively give us £5 from earlier winnings, and we give you the virtual shares in the project. Then what happens is you cash in on these shares at their actual worth-determined directly by the rank of the person and galaxy: in this case, let's say that the project is actually worth £18 - hence you sell the shares at this price and pocket the difference i.e. £18-£5=£13.But I also want to show you how you could lose money- let's say on the other trial, you said a maximum of £16- let's say the market issue was less, actually £14- so the transaction would go through, and you'll pay us £14- but imagine in this case, the actual worth was only £4, you'll end up losing £10. This explains why you should be willing to pay up to a maximum of exactly what you think the shares are actually worth (not more or less)- that way you'll always make a profit when the market price comes up at less than the actual worth, and you'll never lose when the market price comes up above the actual worth- as in the real world of course, it doesn't make sense to want to pay more for something than it's actually worth. And you can also appreciate that it's not a good strategy to underbid in this task as well (explain why if they are not clear). To summarize-you can see you can both win and lose money- the best strategy is to put your maximum price as close as possible to what you think the actual worth of the project is- that way you can only win money in this session.

Any Questions? Ok, so now let me tell you about the other type of trials (“**control”** trials): Again you'll see a person/galaxy pair- and a slider below with a cursor that you need to position. But here you'll notice the scale is different: not a real money scale but instead you see it says: “Person Higher – Same – Galaxy Higher. What you need to do is position the cursor according to whether the person or the galaxy is higher in their respective pecking order: and by how much. For example, if this galaxy has the most amount of mineral, and this person has little power in the company then you should put the cursor over the far right. But if the galaxy is still higher in the order than the person but the difference is less, then put the cursor to the right of the middle but less so. Is that clear? For these trials, you're not stating a price of course, so there won't be any transaction at the end. What we will do, however, is to randomly select one trial at the end of the experiment (exactly as was the case in bid trials), and mark you on how accurate you are, and you will be paid accordingly.
GOOD LUCK!
